# Development of the safety control framework for shield tunneling in close proximity to the operational subway tunnels: case studies in mainland China

**DOI:** 10.1186/s40064-016-2168-7

**Published:** 2016-04-26

**Authors:** Xinggao Li, Dajun Yuan

**Affiliations:** School of Civil Engineering, Beijing Jiaotong University, Beijing, 100044 China

**Keywords:** Shield tunneling, Operational subway tunnel, Key elements of safety control framework for shield tunnel close crossing construction, Remedial grouting, Construction and post-construction settlement of tunnel, Optical fiber measurement

## Abstract

**Introduction:**

China’s largest cities like Beijing and Shanghai have seen a sharp increase in subway network development as a result of the rapid urbanization in the last decade. The cities are still expanding their subway networks now, and many shield tunnels are being or will be constructed in close proximity to the existing operational subway tunnels. The execution plans for the new nearby shield tunnel construction calls for the development of a safety control framework—a set of control standards and best practices to help organizations manage the risks involved.

**Case description:**

Typical case studies and relevant key technical parameters are presented with a view to presenting the resulting safety control framework. The framework, created through collaboration among the relevant parties, addresses and manages the risks in a systematic way based on actual conditions of each tunnel crossing construction. The framework consists of six parts: (1) inspecting the operational subway tunnels; (2) deciding allowed movements of the existing tunnels and tracks; (3) simulating effects of the shield tunneling on the existing tunnels; (4) doing preparation work; (5) monitoring design and information management; and (6) measures and activation mechanism of the countermeasures. The six components are explained and demonstrated in detail.

**Discussion and Evaluation:**

In the end, discussions made involve construction and post-construction settlement of the operational tunnel, application of the remedial grouting to rectify excessive settlements of the operational tunnel, and use of the innovative tool of the optical fiber measurement for tunnel movement monitoring.

**Conclusions:**

It is concluded that the construction movement of the tunnel can be controlled within 15 mm when the shield machine is <7 m in excavation diameter. The post-construction settlement of the tunnel buried in the very soft ground is much greater than its construction settlement, and last several years until reaching a final stable state. Two cases are outlined to demonstrate the feasibility of using the remedial grouting to reduce the long-term settlement of the operational tunnels. The more and more segmental tunnels being constructed, there is an increasing need of the optical fiber measurement for tunnel movement monitoring in the near future.

## Background

As a result of the advancing urbanization in the largest cites of China, huge demand for resources and mobility highlights need for the subway system. Sharp increase in subway tunnel construction has been witnessed in recent years in the cities, particularly the mega-cities of Beijing and Shanghai. Owing to its many advantages such as rapid construction, easy soil deformation controlling, and less disturbances to ground traffic, the Earth Pressure Balance (EPB) shield tunneling method is widely used in constructing subway tunnels in China. In the largest cities, it is inevitable that the new shield tunnels are constructed adjacent to the existing operational subway tunnels during the process of fulfilling or extending the planned subway networks. Moreover, tunneling of utility tunnels and vehicle tunnels in the cities sometimes is accompanied by construction in immediate vicinity of the operational subway tunnels. The new nearby shield tunneling induce ground movement and deformations of the neighboring tunnels, which, if uncontrolled, might not only produce damaging effects to the tunnels but also pose serious threat to people in the tunnels. So all attempts must be made to act upon movement sources of the tunnels and prevent ground decompressions.

The issue of the new nearby shield tunneling has caused widespread concern, and growing attention and interest are focused on it worldwide. Many case histories were reported, such as Samuel et al. (1999) and Cooper and Chapman (1999) both in London, Osborne et al. (2000) in Singapore, Pereira et al. (2009) in São Paulo and Grigoryan (2009) in New York. With the completion of many shield tunnels in close vicinity of the operational subway tunnels in mainland China, relevant construction technologies have progressed rapidly with the accumulated experiences. For example, according to statics the number of the finished tunnel crossing projects was as high as 41 in Shanghai even before 2012 (Zhu et al. 2012). Meantime, many utility shield tunnels were built near the operational subway tunnels. For instance, Shanghai EXPO Beijing West Road to Huaxia West Road power cable tunnel passed the operational subway lines at 12 locations, and shield machines were used to construct the power cable tunnels at the 10 locations (Gao et al. 2009). In the Shenzhen subway 3rd phase project (Lines 7, 9, 11) under construction, new shield tunnels are being constructed in close proximity to the existing operational subway tunnels at 13 locations. Thinking over key elements of the safety control framework for tunnel crossing construction plans in China are still needed on the basis of summarizing and analyzing typical construction cases worldwide.

New shield tunnels constructed in close proximity to the existing operational subway tunnels provide challenges of minimizing movements to the operational systems, and many publications focuses on the challenges. It is particularly necessary to mention that the construction of the North East Line (NEL) presented Singapore with its first such challenge. At two locations, near Dhoby Ghaut and Outram Park Stations, this new line passed 3 and <5 m below the existing operational subway tunnels respectively. Studies were performed to ensure the safe operation of the above existing tunnels. For example, to assess the influences of the tunneling work on the existing tunnels, ten ‘limit states’ were established, with respect to the tunnel structural integrity and track alignment. Shirlaw et al. (2000) described the instrumentation used to check against the ten ‘limit states’, and discussed reporting and assessment of the monitored data. Doran et al. (2000) assessed the total amount of movement that could be tolerated for each limit state after having presented the potential effects of the ten ‘limit states’, five relating to the tunnel structure and trackbed, and five to the subway operation. Osborne et al. (2000) related measures adopted to maintain minimum movements of the existing tunnels, which included a protective pipe roof between the new and the existing tunnels, ground control through the use of an EPB shield machine and extensive on-line monitoring of the ground and the existing tunnels. The first comprehensive study of tunnel and lining response to adjacent tunneling published in the literature was given by Cooper et al. (2002). Among others, Standing and Selman (2001) gave the extensive monitoring within several existing tunnels (District and Circle Line, Bakerloo Line, Shell Center service tunnels Northern Line, Waterloo and City line) mainly in the form of precise leveling, taping and total station surveying of track levels and lining deformations when the Jubilee Line Extension was constructed beneath these existing tunnels. Li and Zeng (2001) studied the influence on one old tunnel, of one new tunnel perpendicularly crossing below the old tunnel, using the 3D finite element method (FEM), and computation results showed advance force and stability ratio were the main factors for controlling deformation of the old tunnel. Kong (2002) described the requirements to be complied by the engineers in the planning, investigation, design and construction of any development within the Railway Protection Zone in Singapore, and precaution/protective measures commonly adopted in the construction control of selected projects, and the behavior of ground and MRT structures observed by various types of instruments during adjacent construction works. Cooper and Chapman (2002) and Cooper et al. (2002) dealt with the monitoring within the Piccadilly Line tunnels during construction of the Heathrow Express tunnels constructed in close proximity to the Central Terminal Area Station, Angel Station as well as Old Street Reconstruction, and a description was given of the instruments used, which included electrolevels, precise levels and tape extensometers, and the relative merits of manual methods and remote reading methods were discussed. Mazek et al. (2003) introduced the construction of the Greater Metro Line 2 and El-Azhar road tunnels under an existing sewage tunnel, and a 3-D model of the multi-crossing tunnel incorporating the effect of grouting was provided to evaluate the usefulness of permeation grouting in reducing deformation around the sewage tunnel when the metro and the road tunnels passed underneath it at different crossing zones. Wang (2003) introduced more than twenty applications of electrolevel beam sensors in the existing tunnels on Lines 1–3 of Shanghai subway to ensure safety of the subway traffic. Shao and Zhang (2004) presented the development of movements of Line 2 tunnels under crossed by Line 4 tunnels of Shanghai subway, and pointed out that the peak uplift always appeared behind the shield tail on account of the action of backfilling grouting. Liao et al. (2005) deduced a theoretical formula of earth chamber pressure of the EPB shield based on tunnel loading model and theory of elasticity, and a so-called ‘step control strategy’ was concluded and applied in building Line 4 tunnels crossing under Line 2 tunnels of Shanghai subway, and the maximum displacement of the existing tunnels was controlled within 5 mm. Li and Zhang (2005) described monitoring and analysis of the tunnel deformations when shield tunnels on Line 4 were constructed under Line 2 tunnels near Dongfang Road Station of Shanghai Subway. Chapman et al. (2006) investigated the use of a simple semi-empirical method for predicting the ground displacements caused by grouting operations, and explained the method, and presented the results as applied to the grouting at Heathrow. Examples of the settlement, rotation and distortions predictions of the Piccadilly Line tunnels made using the methods were compared to the actual measurements, which demonstrated that the method has the potential to provide a reasonable first estimate of the likely effects of grouting on the surrounding ground and, in the case at Heathrow, the effects on an adjacent tunnel. Chen et al. (2006) studied the process of longitudinal settlement of an existing tunnel in Shanghai caused by adjacent shield tunneling on top by combining monitoring data with theoretical analysis. They concluded that (1) when the nearby tunneling is more than 10 m away the settlement caused is negligible, and (2) upheaval during the construction deserves special attention as it may account for almost 69 % of the final movement, and (3) the movement process of the existing tunnel can be divided into four phases: settlement in advance, upheaval during the crossing, short-term upheaval after the crossing, and long-term settlement. Moss and Bowers (2006) presented the running tunnels of the Channel Tunnel Rail Link high-speed railway passing beneath an underground station and under six existing operational subway tunnels, and systematic assessment was undertaken for each tunnel crossing with the capacity of each subway tunnel calculated in terms of an allowable bending curvature, and systems of monitoring trigger levels and associated contingency plans were prepared to manage any adverse events. Wang and Yu (2006) related the construction of Line 2 tunnels of Shanghai Subway above the existing Line 8 tunnels, and gave the recorded response of the existing tunnels. Kojima et al. (2007) described the result of the obstacle paling removal by the Just Cut and Reduce (JC&R) method, and the under crossing of the shield machine when building a road tunnel under the Shinkansen station of the East Japan Railway company. Shao and Zhang (2007) set up a new FEM based on the concept of ground loss, and its applicability was verified by comparing the calculated displacements with those obtained by the empirical method, and the method was used to simulate construction of Line 4 shield tunnels under-crossing Line 2 tunnels of Shanghai subway. Duarte et al. (2009) focused on monitoring of the settlements of Line 2 twin running tunnels during the excavation of Line 4 double-track running tunnel with a shallow overburden of 7.5 m in São Paulo, Brazil. The used on-line monitoring system consisted of an array of precision vibrating wire liquid level settlement sensors. The maximum settlement recorded during the excavation was in the order of 2 mm, yielding a maximum differential settlement of about 1:1900 well below the established tolerance. Fu et al. (2009) presented determination and adjustments of shield driving parameters when building the Shanghai EXPO power tunnel passing under Line 1 subway tunnels based on measured data, and the parameters of earth chamber pressure and simultaneous backfilling grouting were highlighted. Grigoryan (2009) addressed the challenges of designing the underpinning of major subway station with 4 operating tracks during No. 7 subway extension crossing under the existing 42nd street subway station on the 8th Avenue line in New York. Li et al. (2009b) described the risk associated with Line 9 tunnels of Shanghai subway passing above Line 1 shallow buried tunnels, and corresponding countermeasures conducted including shield driving control, ballast weight, reinforcement of the existing tunnels and real-time monitoring. Liao et al. (2009) studied typical construction cases of shield tunnel crossing from above and from below based on field measurements and site investigations of actual projects in Shanghai soft ground, and ground movement prediction and settings of shield driving parameters were demonstrated and summarized in detail. Pereira et al. (2009) presented some considerations about the achieved experience during constructing Line 4 tunnel by the tunnel boring machine under Line 2 tunnels from Companhia do Metropolitano de São Paulo, Brazil. The success of the construction is directly related to teamwork and the great interaction between the professionals of all parties involved in the work (design, construction, tools and operators of the machine). Yang et al. (2009) presented the three dimensional numerical simulation and analysis of Line 10 shield tunnel of Beijing subway crossing under Shaoyaoju station of Line 13. Applications and adjustments of the shield driving parameters together with the monitored performances of Shaoyaoju station were introduced in detail by Li et al. (2009a). Gao et al. (2009) described 10 shield power cable tunnels close crossing the subway tunnels in service or being built or planed. Mohamad et al. (2010) presented a novel technique of distributed strain sensing, whose measured results have close agreement with the data obtained by using total station units, to examine the performance of an old masonry tunnel during the construction of a new tunnel beneath it in London. Chen and Li (2011) presented the method of design and regulation of construction parameters of Line 2 shield tunneling below the existing interval tunnels from Kexueguan station to Dajuyuan station on Line 1 of Shenzhen subway. Chen et al. (2011) presented the measures taken for Shanghai subway Line 7 twin running tunnels passing under the two existing operational Line 1 tunnels at the skew angle of 98° in the EPB shield launching phase. The measures included ground improvement, movement monitoring of the existing tunnels and in-tunnel grouting. Ground improvement zone extended from the usual 3–6 m, and the deep mixing method using cement as a soil stabilizer was adopted. The automatic electrolevel monitoring method was utilized to facilitate the information management. Injecting the two-liquid cement-sodium silicate grout from within the new tunnels was performed to confine further settlement of the operational tunnels. Li and Yuan (2012) reported twin tunnels on Shekou Line of Shenzhen subway were driven by EPB shields under-crossing a double-decked tunnel on Luobao Line, and the performance of the existing tunnel as affected by under-crossing tunnels was monitored and analyzed. Turner and Yap (2011) explored the paradox of commonly used semi-empirical methods for predicting the 3D tunneling-induced ground movements, and investigated the potential effects of the ground movements on the longitudinal and transverse response on the structural behavior of the existing tunnels, and highlighted the shortcomings of commonly adopted assessment methods which may result in non-conservatism and mask critical issues of existing tunnels, and demonstrated the use of modern computing to do powerful complex analysis to solve the complexities of soil–structure interaction and manage risks associated with tunnel crossings. Wang et al. (2011) described Line 6 twin running tunnels of Guangzhou subway crossing 8.1 m under the existing operational Line 1 Huangsha station tunnel along curved lines with minimum radius of 250 m, and measures taken included detailed investigation and assessment of the station tunnel, overall inspection and replacement of disc cutters as well as the automatic monitoring in the existing tunnel. The induced maximum settlement of the station tunnel was about 1.85 mm, which was far less than the alarm value of 4 mm. Yang et al. (2011) analyzed ground disturbance caused by shield crossing base on an above–below crossing project in Shanghai subway construction using the numerical simulation approach, and formulated a series of measures according to theoretical predictions. Zhang and Guo (2011) analyzed the responses of Line 1 tunnels under crossed by Line 3 tunnels of Shenzhen subway using the 3D fast lagrangian analysis of continua method. Zhang and Liu (2011) presented the summary on shield tunnels of Zhujiang Xincheng automated people mover system closely under-crossing Line 1 tunnels of Guangzhou subway. Pan (2011) introduced the construction technologies adopted to building twin running tunnels of Line 11 of Shanghai subway under-crossing the two operational subway tunnels of Line 1 at a skew of 30°, which included selection of shield driving parameters such as earth chamber pressure and advance rate, and the automatic monitoring in the existing tunnels using Electrolevel Beam Sensors. Wang (2011) described the work of injecting the two-liquid cement-sodium silicate grout in existing tunnels on Line 2 of Shanghai subway to confine settlements of Line 2 tunnels caused by constructing Line 11 tunnels. The scheme of using the two-liquid cement-sodium silicate grout to confine settlements and reduce differential settlements of Shanghai subway tunnels in service was put forward (Feng et al. 2011; Xiao and Wang 2011). Zhang et al. (2011) and Zhang and Huang (2014) studied movements of the Shanghai subway Line 4 tunnels caused by the up-line and down-line tunnel on Line 11 crossing under and above respectively, using the 3D finite element analysis program ABAQUS. Huang et al. (2012) studied the effects of the EPB shield tunneling on the above twin existing operational tunnels of Shanghai subway Line 2 on the background of the Shanghai Bund Passage tunnel project using the centrifuge modeling method together with field measurement. In the centrifugal model tests, the influencing factors such as unloading of the excavation, ground loss and grouting were considered to analyze the longitudinal settlement of the new tunnel and the existing tunnels. Besides, the settlement curves of the existing tunnels during the new shield construction were discussed by means of the monitoring data. Ma et al. (2012) studied the effects of the above-crossing construction of a shield tunnel on existing tunnels using centrifuge modelling method on the background of Shanghai’s typical soft ground layers, and the existing tunnels’ behavior and the ground response were analyzed in terms of settlement, pore pressure and internal forces during and after the construction at different grouting ratios. Centrifuge tests showed grouting can alleviate the heave of the existing tunnel. However, excessive grouting ratio during construction will disturb the surrounding ground and cause more settlement in the long run after the construction. Yuan (2012) gave the monitoring plan, the shield driving control and the in-tunnel loading measures when building Shanghai bund passage tunnel about 1.4 m above the two existing operational Line 2 tunnels of Shanghai subway. The Shanghai bund passage tunnel with the overburden of only 7.9 m at the crossing location was excavated using a 14.27 m-diameter EPB shield. The measured maximum uplifts of the up-line and down-line tunnel were 10.90 and 10.14 mm respectively. Zhu et al. (2012) analyzed three factors influencing the crossing construction in Shanghai: the vertical interval between the existing tunnel and the new tunnel, the projection angel and the synchronous grouting rate, and obtained linear regression equations to show the trends of displacement during the overcrossing and the undercrossing construction projects. Han (2013) introduced the construction technology and measures taken for constructing the Shanghai Expo power cable tunnel passing 9.4 and 3 m under the twin operational Line 4 tunnels of Shanghai subway. The technology centered on selection of the EPB shield driving parameters and backfilling grouting in different crossing stages. Jiang (2013) described the technologies, including obstacle exploration in advance, consolidation of ground between the shield tunnel and the existing subway station, determination of proper shield tunneling parameters and grouting parameters, optimization of the grouting system of the shield machine, and particularly the injecting bentonite into the gap between the excavation and the shield shell before performing the simultaneous backfilling grouting, adopted in constructing twin running runnels of Line 8 2nd phase project 2.5 m under the existing operational Guloudajie station of Beijing subway Line 2. The settlement of the station tunnel induced by the shield tunneling was <1.7 mm, not exceeding the prescribed tolerance. Zhang et al. (2013) studied the internal force and movement of Suzhou subway Line 1 tunnel under-crossed by a new shield tunnel using the 3D FEM, and analyzed influences of the spacing between the two tunnels. Zhang and Zhang (2013) studied deformation prediction of Line 4 twin operational tunnels of Shanghai subway caused by Line 11 up-line tunnel 1.82 m under-crossing and down-line tunnel 1.69 m above-crossing using the simplified theoretical method, 3D FEM simulation, and in situ monitoring method. Adopted EPB shield driving parameters, such as earth chamber pressure, annular filling grouting, and shield advance rate and cutterhead torque were also detailed. Chen et al. (2014) introduced measures and countermeasures adopted in shield tunneling under the existing operational subway tunnels in Shenzhen. The existing tunnel near the shield launching shaft, a special steel sleeve used for the launching was fixed at the tunnel portal, and such measures and countermeasures as ground improvement, adjustment of shield driving parameters and information feedback construction were also employed to ensure safety of the under-crossing. Fan (2014) described the schemes for constructing twin Line 3 west extension tunnels of Shenzhen subway, 1.23 and 1.46 m under the two existing operational subway Line 1 tunnels respectively. The adopted technologies were centered on shield driving parameter selection, backfilling grouting and in-tunnel monitoring, and injecting bentonite around the shield. In this project, limiting the speed of trains <25 km/h during the construction was employed for sake of the public safety. Li et al. (2014) described the shield tunneling technology and measures taken when Line 11 twin running tunnels of Shenzhen subway crossing 1.5 m above Line 1 twin existing operational tunnels. Improving ground above the existing tunnels, elaborate shield driving control together with in-tunnel automatic monitoring helped control the uplift of the existing tunnels within 2 mm and guarantee safety of the subway traffic. Wang et al. (2014) used the 3D FEM to simulate the excavation process of three crossing forms of the twin new tunnels: first above and then under crossing, two under crossings and two above crossing, based on the actual soil layer of one tunnel overlapping section of Shanghai subway. Reliability of the numerical simulation was verified by the comparison between measured and simulated data. Settlements of the existing tunnels and measures for controlling the settlements were given in the each crossing form. Yun et al. (2014) developed a Principal Component Analysis (PCA)-based monitoring framework using sensor data collected from the existing tunnel while the new tunnel was excavated, which is particularly useful to analyze underdetermined systems due to insufficient sensor data for explicit relations between force and deformation as the system input and output respectively. Zhang et al. (2014) analyzed influence of the two new tunnels construction on the existing twin tunnels for two construction methods of above-crossing and under-crossing, using the commercial program (ABAQUS), and a modified Gaussian equation was proposed to predict ground settlement according to the computation results. Results showed that a secondary disturbance was to be caused by the shield advance, and greater influences would be exercised on the existing tunnels when the new tunnels were closely spaced. In addition, many other similar shield tunnel close crossing construction projects in mainland China, not reported in literatures or official publications due to various reasons, were realized in demanding conditions not only in respect of inner city environment where space requirements had to be taken into regard but also in respect of safe operations of the neighboring subway tunnels. For instance, in Hangzhou the first shield tunnel crossing project was finished in December 2013, of Hangzhou subway Line 4 tunnel between Guanhe station and Railway East station passing 2.1 m below the twin operational Line 1 tunnels after about 29 m driving from the launching excavation. The Hangzhou subway Line 4 interval tunnel between Qianjianglu station and Jiangjinlu station passing about 1.273 m below Line 1 tunnels was successfully accomplished on April 15, 2014. It is obvious that uttermost publications presented above dealt with only a single case study mainly because of the tribal knowledge of the involved projects. Generalized studies from the many case studies in combination with some undocumented recordings are necessary to improve the shield tunneling in close proximity to the existing operational subway tunnels. Moreover, it is often necessary in practice to take into consideration a set of distinct elements that frequently influence the construction of tunnels near the existing subway tunnels. Then well-targeted graduated response plans are designed to systematically and consistently manage safeties of the projects throughout the whole construction process. Although further studies on some topics do necessitate performing, a common understanding and recognition of the safety control framework has been reached in many aspects of the shield tunneling in close proximity to the existing operational subway tunnels in mainland China. Six key elements of the framework are presented. The conclusions of the elements, on which a consensus can be reached, are generalized from the typical close crossing construction projects in mainland China, while individual differences are illuminated. The purpose of the paper is to give the state-of-the-art review on the key elements of the safety control framework for shield tunneling in close proximity to the operational subway tunnels, so as to provide references for later similar projects.

## Construction method and shield tunnel close crossing schemes

### Choice of the new nearby tunneling method

The existing subway tunnel operation requires that to put the maximum effort in reducing, as much as reasonably possible, the occurrence of excessive movements of the existing tunnels is a ‘must’. Therefore, the first step of the approach to success even at a higher price, when tunneling adjacent to the existing operational tunnel, is choosing the reasonable construction method together with implementing whatever measures necessary to realize the safe operation of the existing tunnel. In uttermost construction cases of mainland China the shield tunneling method with pressurized face support is required and adopted in view of its many advantages such as the rapidity and industrialization of the construction cycle with possible automation, the possibility to measure and keep under control the principle construction parameters and the guaranteed quality of the finished work using precast segments to line the tunnel. For example, a section of a power cable tunnel under-crossing Line 1 tunnel of Beijing subway was constructed using the shield tunneling method whereas the remaining part was constructed by manual excavation, even if the improved mining method is verified to be suitable for ground conditions of Beijing area. Also the shield tunneling technology offers a high degree of planning certainty in terms of both finance and time compared to the conventional techniques. Moreover, a challenge of the future will be to keep up mobility of people and goods and data, protection of the surroundings and availability of resources. The shield tunneling method offers the technology to meet the challenge both for traffic and utility tunneling.

### Crossing schemes for the new nearby shield tunneling

The single track tunnel is widely adopted in subway systems of mainland China, and usually twin single-track tunnels connect neighboring subway stations. The impacts of the twin tunnels construction on adjacent operational subway tunnels have something to do with the twin tunnels interactions, which depend to a great degree on the spacing between the tunnels. In most cases the spacing is not too large due to limitations of the underground space. The tunneling of the twin tunnels may be concurrent or totally staggered. When the twin tunnels are constructed concurrently, the interactions are strong and may cause the larger movements of neighboring tunnels. The almost concurrent construction of the Jubilee Line Extension (JLE) under the Bakerloo and Northern Line tunnels were presented by Standing and Selman (2001). Compared with the scheme for concurrent construction of the twin tunnels, the scheme for staggered construction of the twin tunnels is a better choice in most cases and has the advantages (Li and Yuan 2012):

1. The twin tunnels being built over different periods not concurrently, the likelihood of the neighboring existing tunnel to suffering from disaster is greatly reduced, and the risk management of the subway tunnel operation is easy to fulfill and realize.
2. Over the rest period before the second close crossing, ground improvement can be employed to increase the stiffness of the surrounding ground in which the second tunnel is excavated, thus decreasing the second excavation induced ground movements. For instance, In a project of twin shield tunnels passing beneath a subway station structure on ground surface presented by Li et al. (2009a) and Yang et al. (2009), ground improvement underlying the surface structure performed over the period of close to 1 year before the second excavation, together with the selection of more reasonable shield tunneling parameters, helped reduce the maximum settlement of the overlying structure from 15.3 mm of the first excavation to 4.6 mm of the second excavation.
3. Information gathered and experiences gained in the first close crossing are valuable for the second crossing, which can be fully utilized to reduce the impacts on the existing tunnels in service.
4. The staggered construction is sometimes more economical, for one shield machine is enough to finish the two crossings of twin tunnels if the construction time limit permits.

In view of the above merits, the staggered scheme was widely used in construction of the subway tunnels in China. For example, the staggered crossing scheme was applied to building twin shield tunnels on Shekou Line of Shenzhen subway crossing under the double deck tunnel on Luobao Line, and the first crossing induced settlements of the above tunnel had already tended to be stable before the second crossing (Li and Yuan 2012). In the project of Line 2 tunnels under crossed by Line 4 tunnels of Shanghai subway, the second under-crossing was performed more than 3 months later (Wang et al. 2004; Hu and Huang 2006). In the project of Line 2 tunnels above crossed by Line 8 tunnels of Shanghai subway, the second under-crossing was performed nearly 2 months later (Wang and Yu 2006).

## Key elements of the safety control framework

The movement of the existing tunnel induced by the new nearby shield tunneling is decided by conditions of the surrounding ground and existing tunnel as well as methodologies and auxiliary measures to be utilized. There always being unknown information concerning these factors, a flexible design of the safety control framework is often adopted, in order to be prepared to face unforeseen and unfavorable situations. Attention is paid to the critical elements and relevant works of the framework.

The framework centers on the safety control of the operational subway tunnels. The safety of the operational tunnels must be guaranteed when new shield tunnels are constructed in the immediate vicinity. To realize this aim. The conditions of the existing tunnels should be mastered to the largest degree. So an overall and thorough inspection to investigate the conditions were always performed beforehand. To guide the new nearby shield tunnel construction, it is a must to decide the allowed movements of the existing tunnels and tracks. To help make the execution plans, it is necessary to simulate effects of the new shield tunneling on the existing tunnels by numerical computations. The preparation work must be done in advance and can be done well with the findings of the computations. Whereas monitoring design and information management are indispensable to the information-oriented new nearby shield tunnel construction. Referring to the monitored results, measures and countermeasures can be taken when necessary. In accordance with the above ideas, the developed framework is the result of a collaboration among the involved parties. Key elements of the safety control framework, identified for a fully integrated design and construction procedure and based on the most recent and state-of-the-art experiences in mainland China, can be summarized and given below.

### Inspecting the existing operational subway tunnels

Successful shield tunneling near the existing subway tunnel requires correct understanding of the surrounding environment made up of infrastructures on the surface, above-surface and subsurface utilities, of the natural ground and the existing tunnel. The first three elements, may be, to a certain degree, documented and can be investigated with relative ease. More exploration holes may be bored at the construction site to understand the ground conditions if ground surface permitted. The last element is of uttermost importance because of the allowed movement of the existing tunnel heavily dependent on its condition. However, the work of the in-tunnel investigation is a piece of work on account of the subway tunnels in use in the daytime, and it can only be performed at night when the subway traffic is halted and the in-tunnel power supply switched off.

Subway tunnels are not only structures constructed of reinforced concrete, but also use numerous functional systems, such as track, signals, communications and power supply. Before in-tunnel investigations, the design and construction documents as well as the maintenance records of the tunnels are reviewed, and relevant important information collected is listed below:

1. The construction method of the existing subway tunnel;
2. The cross section, material and structure of the existing tunnel;
3. The track structures in the existing tunnels;
4. The in-tunnel power supply system;
5. Other involved systems/appurtenances.

**Fig. 1:**
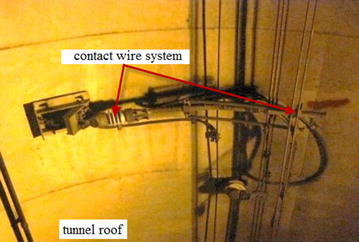
Flexible overhead contact wire system installed at tunnel roof

The safety of the train in a subway tunnel is closely related to deformations of the track structures, and particularly the type of fastener, the gauge and rail surface adjustment must be mastered in advance of tunneling. The power supply system installed in the tunnel deforms along with movements of the existing tunnel, and it’s allowed movement has something to do with the speed of the subway traffic. Taking the flexible overhead contact wire system used in some cities of mainland China for example (see Fig. 1), the allowable slope of the contact wire varies with the train speed, as prescribed in Chinese Code for design of subway (GB50157-2013) and shown in Table 1. Therefore, the allowable movement of the existing tunnel is influenced by speed of the in-tunnel trains, and limiting the speed in most cases is the most useful measure to guarantee safety of the running trains. A thorough investigation of the involved systems/appurtenances is indispensable to realizing safety of both the tunnel close crossing construction and the subway system operation.

**Table 1:** Maximum slope of contact wire

Train speed (km/h)	Maximum slope of contact wire (‰)
10	40
30	20
60	10
90	6
120	5

**Fig. 2:**
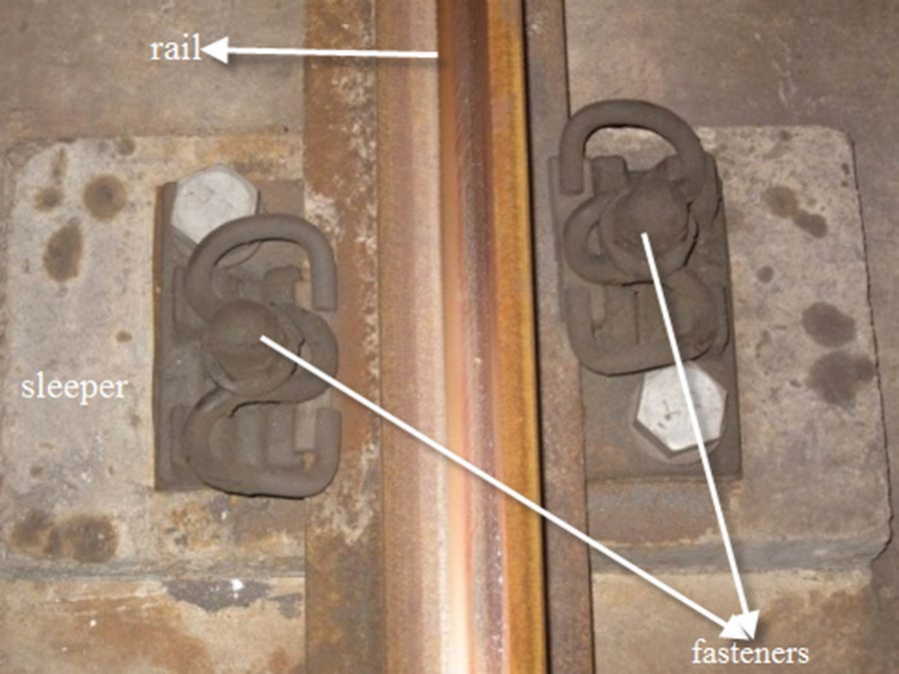
One type of fastener used in subway tunnel

**Fig. 3:**
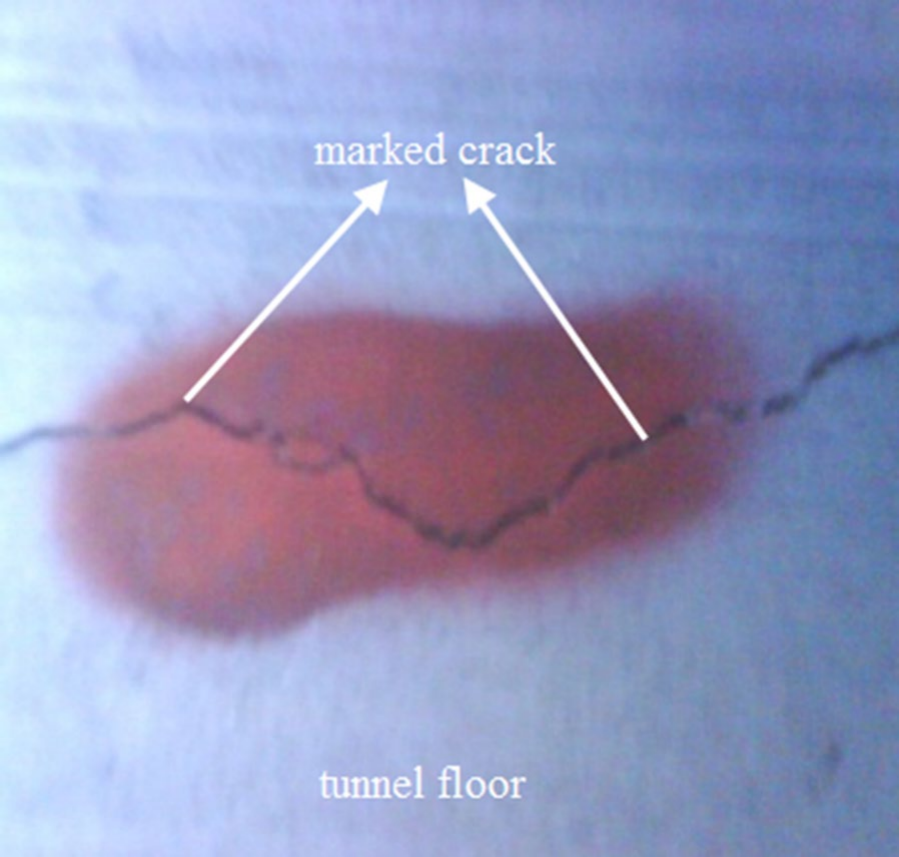
Crack in subway tunnel structure

**Fig. 4:**
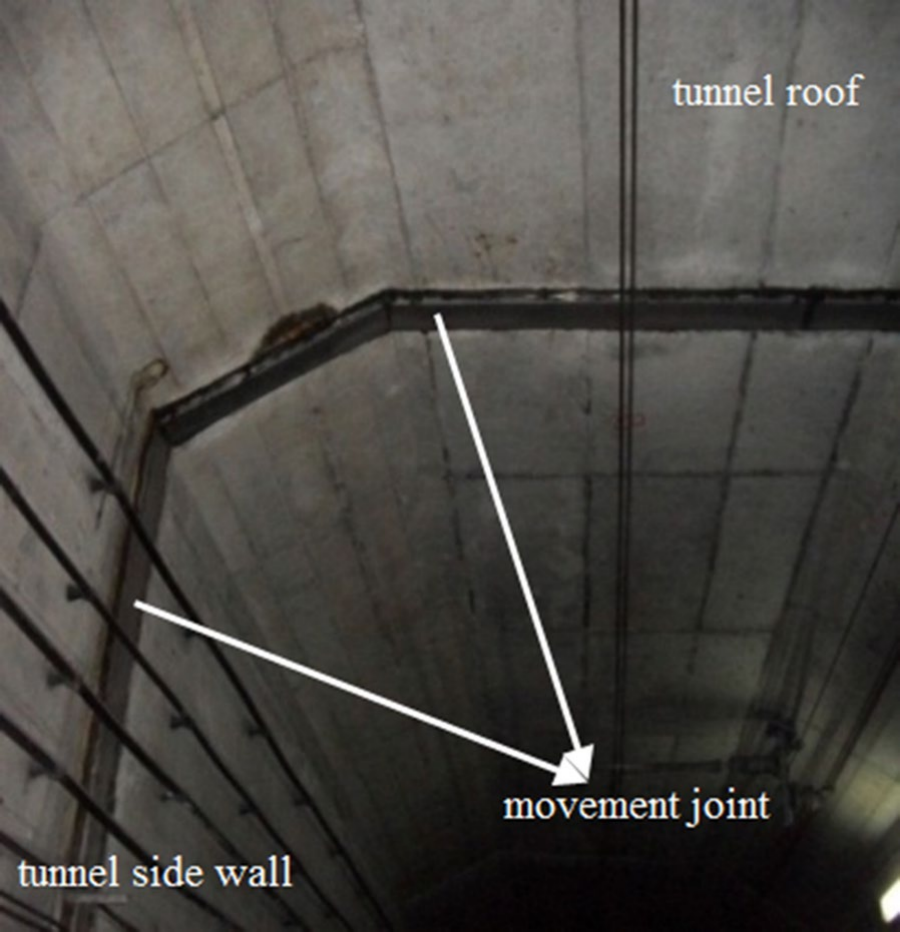
Movement joint in subway tunnel structure

To aid the information-oriented shield tunneling a thorough in-tunnel investigation must be conducted in advance of tunneling to verify the gathered data, especially information of the fastener (for example, see Fig. 2), and to inspect condition of the subway tunnel structure within influence zone. Especially the information of the cracks (for example, see Fig. 3) and movement joints (for example, see Fig. 4) in tunnel structure should be well recorded, and the location, width, extension of the cracks should be marked out in order to easily recognize their changes. For a segmental tunnel, types of segments, joints and gaskets are all important information. The abnormal segment opening must be labelled and recorded for special attention lest water leakage into subway tunnels should occur.

In mainland China, the operation, maintenance, and inspection of a subway tunnel are thoroughly regimented to provide an adequate level of safety. With the limited access and confined conditions within an affected subway tunnel, inspection and maintenance of the tunnel are conducted under the supervision of the tunnel administrator/owner. This is particularly the case in the shield tunnel close crossing projects. Before staring crossing, maintenance and inspection are closely intertwined to provide a proactive approach to sustaining an operating subway tunnel. During the crossing process, maintenance and inspection, scheduled and contemplated on a daily basis, are closely coordinated at least to ensure the normal running of the in-tunnel trains by correcting movements of the rails induced by the new nearby shield tunneling. To deal with the limited situation, automatic maintenance and inspection may be employed without interruption to normal subway traffic by robots equipped with the advanced technologies.

### Deciding allowed movements of the existing tunnels and tracks

Deciding allowed movement of the operational subway tunnel and track is the key to realize safety of the subway traffic operation. Safety indexes for tunnel close crossing construction are expressed not only in terms of movement of the existing tunnel but also deformation of the in-tunnel track, and both of them must be satisfied simultaneously.

1. Allowed movement of the existing tunnel

Although the safety of tunnels as affected by adjacent shield tunneling activities has been a main concern in mainland China underground space utilization, determining the allowed movement of a damaged tunnel in service is an extensive, wide-ranging and challenging task requiring an interdisciplinary effort, and hasn’t been well solved in theory up till now. The difficulty of the task lies in three aspects: (a) the impossibility of collecting all the imperfections in tunnel structures; (b) the perplexity of understanding the relationship between the condition of a tunnel and its deformation resistant capacity and (c) the complexity of the natural surrounding ground. For application in practice, criteria have been attempted to establish to restrict the induced movement of the existing tunnel. Because of the lack of case histories on damage to tunnels, most of the criteria adopted were mainly based on theoretical considerations and plausibility, and were more or less arbitrary. A better suggestion is to investigate separately the shield tunneling induced movements of the existing tunnels in each city. Firstly, an in-depth analysis is performed to find the key factors influencing movements of the existing tunnels. Then the relationship between the tunnel movement and its influencing factors can be set up by using the regression analysis method. With the gotten equations, the allowed movement can be determined with more objectivity. Of course, one important premise for the suggestion is the many similar projects finished.

For example, the established criteria in Shanghai area early in 1994 and later used for reference by other cities, such as Shenzhen, are as follows (Wang and Liu 2004):

- The maximum settlement and horizontal displacement—no more than 20 mm;
- The radius of deformation curve along the longitudinal direction of the existing tunnel—no <15,000 m;
- The slope of deformation curve along the longitudinal direction of the existing tunnel—no more than 1/2500.

The above criteria were employed in some tunnel close crossing construction projects (Liu et al. 2005) in Shanghai as well as some projects in Shenzhen, including a double deck tunnel and two running tunnels on Line 1 under crossed by tunnels on Line 2, tunnels on Line 1 under crossed by tunnels on Line 3, and tunnels on Line 2 under crossed by tunnels on Line 4. The Shanghai soft ground is composed of littoral deposits featuring with saturated, flow to soft plastic clay with high compressibility and sensitivity and low strength, long stabilizing time and big settlement after being disturbed. The existing subway tunnels often have already produced some even large settlements on account of the long-term consolidation settlements of underlying soil (Wang and Liu 2001; Ye et al. 2007). In some later projects, more strict criteria were adopted. The allowed settlement and uplift were both reduced to 5 mm and the demand for tunnel convergence was <20 mm in projects of Shanghai, embracing tunnels on Line 2 under-crossed by tunnels on Line 4 (Wang et al. 2004; Hu and Huang 2006), tunnels on Line 2 above-crossed by tunnels on Line 8 (Wang and Yu 2006), and others presented by Chen et al. (2009) and Zhu and Huang (2010).

In Beijing area, the case-by-case consideration was required considering that no two close crossing projects were exactly alike and especially when involved were station tunnels or tunnels sensitive to surrounding ground movements such as the very old tunnels on Line 1 of Beijing subway, which were open to public in 1969. The allowed movement of a subway tunnel was decided by invited experts on the basis of enthusiastic discussions and analysis, usually the decided criteria of tunnel movements were in the ranges of 5–10 mm.

In the two lately finished projects of Line 4 shield tunnels passing in two locations below the existing operational Line 1 tunnels in Hangzhou, the allowed vertical movement (settlement and uplift) and horizontal movement were both limited within 5 mm and the segment lining convergence should be <5 mm.

In the project of Line 6 twin running tunnels of Guangzhou subway crossing 8.1 m under the existing operational Line 1 Huangsha station (Wang et al. 2011), the allowed settlement of the tunnel structure was less than and equal to 10 mm, and the attention and alarm values of the horizontal movement and settlement were 4 and 6 mm respectively.

Moreover, it is fully understood that maximum displacement is not a governing factor in evaluating the safety of the subway tunnels while it is the curvature of the settlement or uplift profile that matters. However, from a practical point of view, maximum displacements are certainly important indexes in evaluation of the safety of a tunnel. In most tunnel close crossing construction projects of China, only the criteria of the maximum movements were employed and executed.

It is worthwhile to point out that in almost all tunnel close crossing projects experienced engineers were assigned to inspect developments of the cracks in the existing tunnels at every night over the whole period of crossing construction aiming at testifying the validity of the decided allowed movements of the existing tunnels.

(2) Allowed movements of tracks

The track structure in most subway tunnels of mainland China is made up of the monolithic track bed, concrete bi-block sleepers, rail fasteners, and the connected rails. The adopted indexes for tack movement criteria are horizontal alignment, vertical alignment, twist, as well as track gauge of the rails and movement of the track bed. It is favorable for the tunnel close crossing construction projects that the rail surface can be adjusted to ensure the smooth running of the trains by inserting and removing the underboarding under the rails at night, if large movement of the track bed is induced by the nearby construction. So the permitted movements of the tracks are linked with the daily maintenance and types of the used rail fasteners. Given in Table 2 are some rail fasteners used in Beijing subway (Li 2010). Considering construction errors and leaving certain allowance, usually 50 % of the adjustment of the rail fastener was taken as the daily allowed settlement of the track bed.

**Table 2:** Adjustments of some Rail fasteners used in Beijing subway (unit: mm)

Type of rail fastener	Adjustment of track gauge	Horizontal adjustment	Adjusting height under rail	Adjusting height under tie plate
DT I	8, −12	10	0	10
DT III	8, −12	25	10	15
DT IV	8, −12	25	10	15
DT V	8, −12	15	0	15
DT VI	8, −12	15	0	15
DT VI1	4, −8	30	0	30
DT VI2	8, −12	30	0	30
DT VII	8, −16	25	10	15
DT VII2	8, −16	40	10	30

Shown in Table 3 are the allowed deformations of rails, which are connected with the thresholds for different maintenance plans. Over the period of tunnel close crossing construction in Beijing, the daily allowed deformations of rails are usually 30–50 % of the threshold values for the planned maintenance. At night and after subway traffic halt, once the measured behaviors of rails exceed the daily allowances, the repairing work is implemented to restore the track geometries within the allowed ranges.

**Table 3:** Thresholds for maintenance of some lines of Beijing subway (unit: mm)

Type	Item	Planned maintenance		Regular maintenance	
Line	Gauge	Main line	Other line	Main line	Other line
	Horizontal	+4, −2	+5, −2	+6, −3	+7, −3
	Vertical	4	5	6	8
	Direction (line)	4	5	6	8
	Twist				
	Transition curve	4	5	6	8
	Branch line and circular line	4	5	6	8
Turnout	Gauge				
	General place	+3, −2	+3, −2	+4, −3	+4, −3
	Sharp end of switch rail	3	4	5	7
	Horizontal	3	4	5	7
	Vertical	3	4	5	7
	Direction				
	Line	3	4	5	7
	Offset	2	2	3	3

In Shanghai the specified limits for track alignment are: gauge widening less than and equal to 3 mm, gauge narrowing less than and equal to 1 mm, horizontal alignment less than or equal to 2 mm, top (vertical alignment) and twist <2 mm/10 m (Wang and Liu 2004; Hu and Huang 2006).

In Shenzhen the required limits for track alignment and adopted in recent under-crossing projects are: horizontal alignment less than or equal to 4 mm, top (vertical alignment) less than and equal to 4 mm/10 m and twist less than and equal to 4 mm/18 m.

In a shield tunnel crossing construction project of Guangzhou subway, the allowed track geometries were: horizontal <4 mm, track gauge movement in +6 to −2 mm and vertical <4 mm over 10 m chord (Wang et al. 2011).

### Simulating impacts of the new nearby shield tunneling on existing tunnels

By means of 3D FEM or FDM, the response of the operational subway tunnel to neighboring shield tunneling has been rigorously assessed both in normal and anomalous conditions, i.e. considering a set of potential scenarios at the design stage in almost all close tunnel crossing construction projects of mainland China (such as Yang et al. 2009). Main construction phases simulated step by step, key parameters to be observed and regulated such as the face support pressure and grouting volume, can be identified and selected with the numerical simulations. At the same time, the most critical aspects of the tunnel close crossing can be understood by simulating the possible crisis scenarios and defining a course of countermeasures for every predictable significant deviation of the observational findings from those predicted on the basis of the predefined working hypothesis. On account of the above characteristics of the numerical calculations, it was required that impacts of the shield tunneling on existing tunnels were simulated using the 3D FEM or FDM. But it is necessary to point out that the computation quantitative results of the FEM/FDM weren’t taken as absolute values owing to calculations based on a variety of assumptions, simplifications of some construction details as well as inaccurate selection of parameters and constitutive equations of soils. A suggestion is to back-analyze the shield tunneling induced movements of the existing tunnels. Using the back-analysis method, reasonable parameters and models to describe the complex interaction between tunnel and ground may be gotten in each city. By combining the findings with the computation by the FEM/FDM for a new nearby shield tunnel construction in this city, the calculated results can be gotten with more reliability. The findings by the FEM or FDM do provide important references for making the execution plans. Measures and countermeasures can be taken against the worst scenarios in construction according to the findings. It is a necessary part of the framework. The findings by the calculations do provide crossing projects with the directional guidance in construction, and at least can be employed as a basis for qualitative judgment. It is encouraging that the simulation precision is increased solidly with the more tunnel close crossing projects completed and more numerical calculations performed in the largest cities of China.

### Doing preparation work

The shield tunneling in close proximity to the subway tunnels in service is a system work and many aspects of facilities, materials, workers, workmanship and etc. are involved and overall preparations are needed before starting crossing. Only the crucial jobs are highlighted herein.

1. Zoning management of the tunnel close crossing construction projects

There being the limitations for applications of the FEM and FDM to simulating construction of the new nearby shield tunneling, some details and key parameters of the shield tunneling can never be realistically modeled. Accordingly, a test zone was often delimited before starting crossing to understand the relationship between the shield tunneling parameters and the surrounding ground disturbances, for conveniences of construction management as well as accumulating experiences of controlling the shield machines. The zone between the ring of six segmental rings width in front of the existing tunnel and that of six segmental rings width behind the existing tunnel was always defined as the crossing zone in case of perpendicularly crossing. While in case of the oblique crossing, the projection length of the new tunnel onto the existing tunnel was used to determine the crossing zone. The range of 20–30 segmental rings width in front of the crossing zone was regarded as the test zone (Zhu and Huang 2010; Chen and Li. 2011).

(2) Suggesting values for key parameters of the shield driving

In favor of the construction planning, reasonable ranges of the parameters are suggested for application in the crossing construction. The parameters include earth chamber pressure, jack thrust, advance rate of shield machine, rotation and torque of cutter head, discharged volume of the excavated soil, mix ratio and injection volume of ground conditioning agent such as form, as well as mix proportion, grouting pressure, and grouting volume of simultaneous backfilling grouting, based on shield tunneling in the test zone (Chen and Li 2011). Of course, suggested values could be adjusted anytime in light of performances of the shield driving and measured movements of the existing tunnels.

The relationship among important shield tunneling parameters and its effects on the existing tunnels still remain an issue. Usually the existing tunnel will move toward the excavated space. The existing tunnel will settle in case of under-crossing and rise in case of above-crossing. But this is not always the case. For example, in some under-crossing projects the existing tunnels rise due to adopting the higher earth chamber pressure and the rapid advance rate. The phenomena are also relevant to with the soft ground of Shanghai area for they have not been observed in other cities of China up till now. Further study and efforts are still needed to set up the quantitative relations between shield tunneling parameters and their effects on existing tunnels.

How to realize the shield tunnel close crossing construction can be learned from previous construction experiences. Especially the principles of slow driving, making an even turn, keeping enough face supporting pressure and a reasonable grouting, are popularized in the tunnel crossing construction projects of Shanghai. During Line 4 tunnels under and curved crossing Line 2 tunnels, the parameters adopted were the advancing rate <10 mm/min, the tunnel axis error <10 mm, the horizontal deviation-rectifying <20 mm per ring and the vertical deviation-rectifying <0.05° per ring (Hu and Huang 2006). In another above and curved crossing project of Shanghai subway, the advance rate was required to be <20 mm/min (Chen et al. 2009). In other cities such as Beijing and Shenzhen the advance rate of shield machines in the crossing zone is often required to be <20 mm/min.

(3) Preparations for the secondary phase grouting work of the shield tunneling

**Fig. 5:**
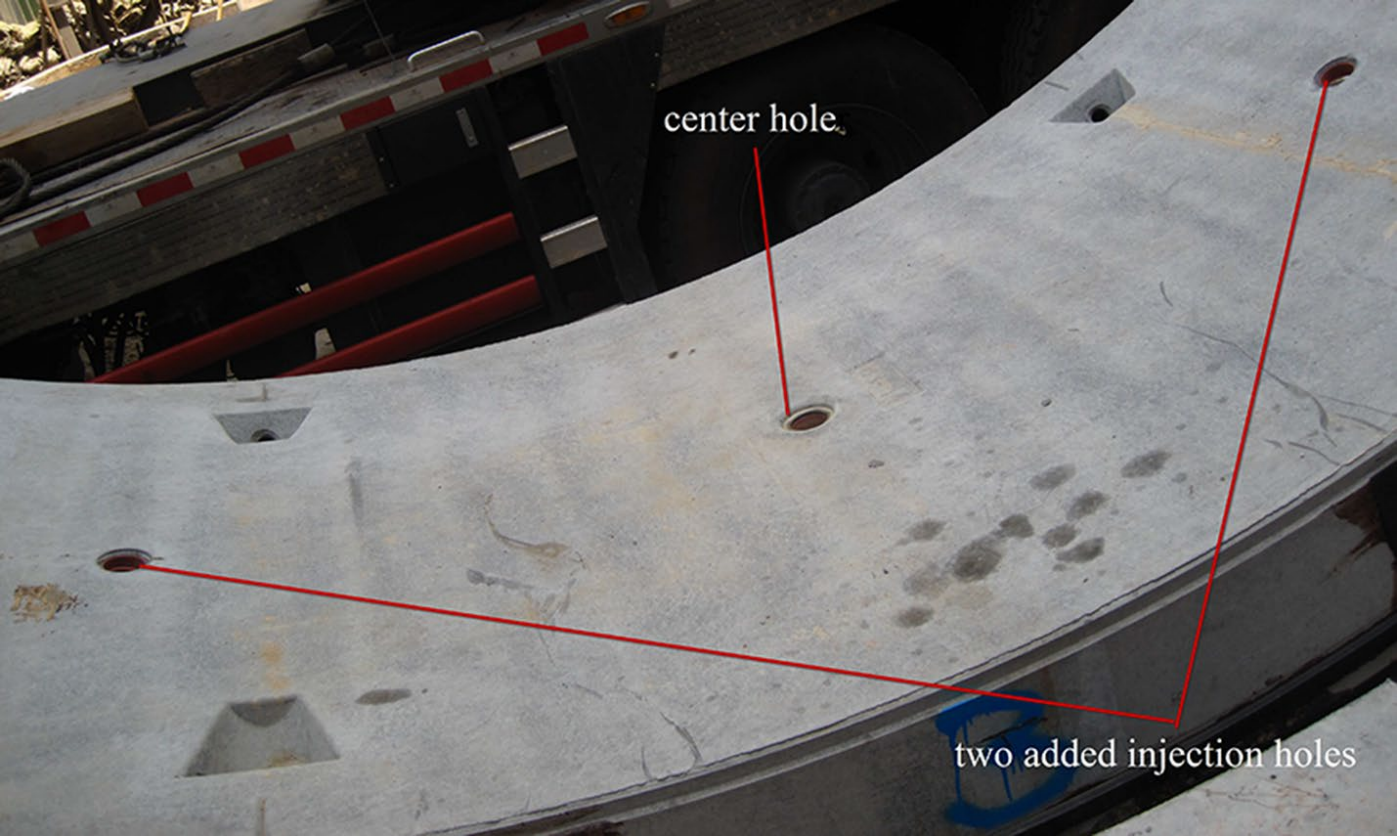
A center hole and two added injection holes

It is necessary to perform overall preparations for workmanship, materials, equipment and etc. involved in the secondary phase grouting work of the shield tunnel close crossing construction projects (Li and Chen 2012). Herein emphasized is the preparation for other injection holes in a segment and special designed mixer for injections through segments (Li and Chen 2012). Usually the center hole of a segment for the erector can be used as the injection hole but in order to carry out the injection as early as possible in the back-up system of the shield machine, other preformed holes in the segments can be prepared in the segment factory (see Fig. 5). In one segment ring other injection holes are added and this will undoubtedly increase the convenience and more freedom for performing the grouting work. In many construction cases, early solidification of the used two-liquid type grout of cement-sodium silicate increases chances of blocking grouting pipes; therefore a reasonably designed mixer is of special importance for the application of the injection. This work was often finished before starting crossing.

### Monitoring design and information management

The design of the monitoring system is one of the central elements of the construction plan for tunnel crossing construction projects, together with active recording and interpretation of the shield tunneling parameters. To respond effectively the complex situations such as collapse and too large settlements of the existing tunnels, and realize a fully integrated design and construction procedure, the combination of the automatic remote reading and manual method can meet the requirements, as already explained by Cooper and Chapman (2002). In mainland China, the following components have been identified for a comprehensive and effective design of a monitoring system.

1. In-tunnel monitoring

Controlling movement of the existing tunnel is heavily dependent on the accurate and timely information, as well as the ability to use this information effectively. Therefore, there is a clear demand for real-time monitoring and availability of results to check and detect any anomalous trend in the monitored parameters and manage any unforeseen events when tunnels are constructed near the existing operational subway tunnels. On the other hand, reasonable values of the shield tunneling parameters are crucial to reducing impacts on operation of the subway tunnels. To aid the entire information oriented process of the tunnel close passing projects, adjustments of the shield tunneling parameters are made based on the measured results. The real-time monitoring of relevant construction parameters is a necessary part of shield tunnel close crossing construction projects.

The in-tunnel automatic high-performance remote reading method, like the in-tunnel monitoring in Singapore and London (Sharma et al. 2000; Cooper and Chapman 2002), are widely adopted in tunnel crossing construction projects of China; different from the instruments used in London, the monitoring system being used in China can be classified as three types, i.e. 3-D total station measuring system, hydrostatic leveling system and electrolevel beam sensors measuring system. As to other fundamental monitoring program components such as layout of measuring points, measuring frequency, they were usually decided as needed. The extent of the monitoring covered width of the predicted movement trough of the existing tunnel. As to the frequency of monitoring, remote readings were made once per 15–30 min when shield tunneling in the crossing zone. Manual monitoring was executed at night when trains stopped. Base reading were often taken 1–2 months in advance of the crossing construction.

**Fig. 6:**
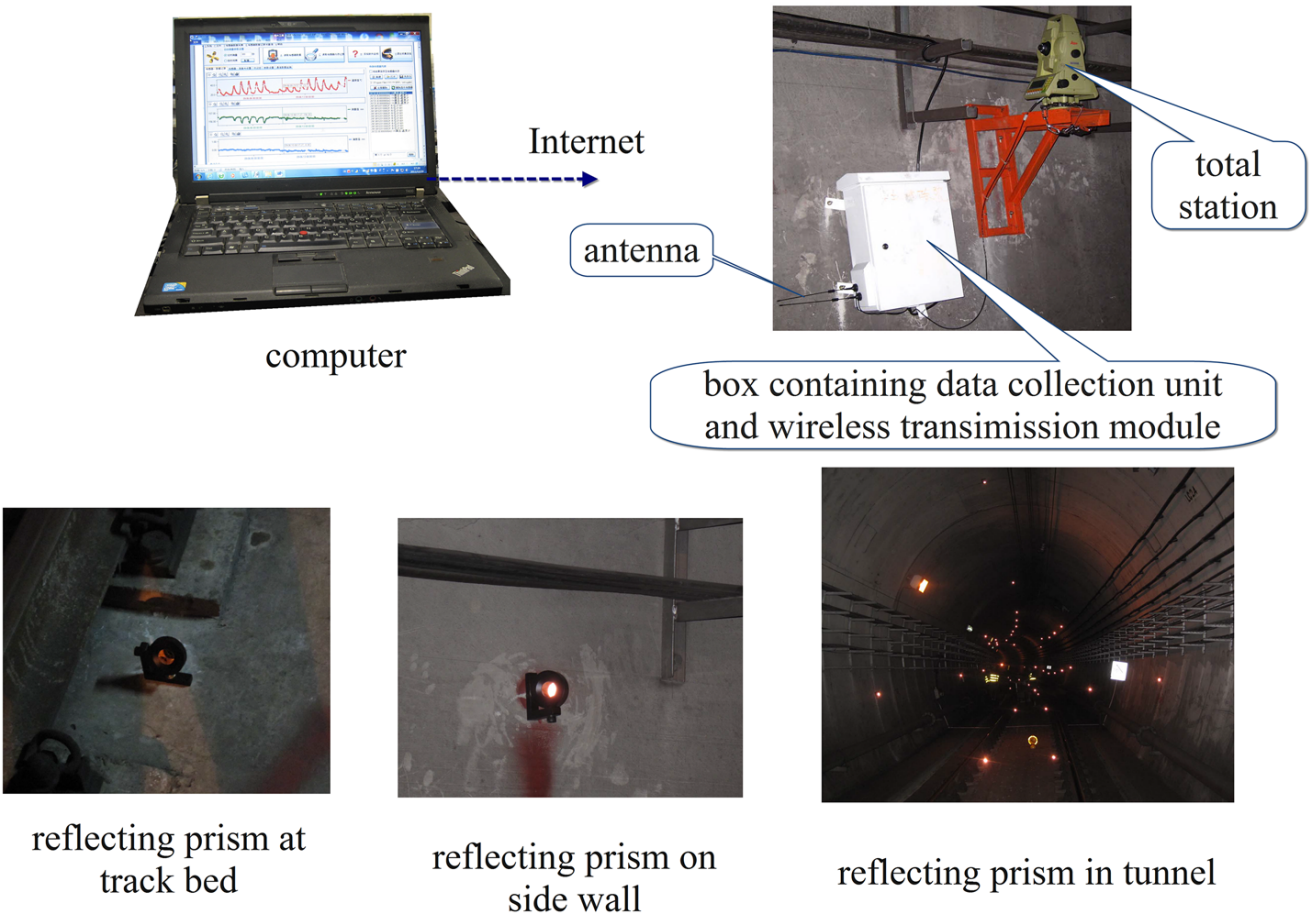
Total station measuring system

Shown in Fig. 6 is the Leica TCA2003 total station measuring system, made up of the total station, the Code Division Multiple Access (CDMA) wireless transmission module in a box, the antenna, the built-in GeoMos software and the reflecting prisms. The total station system is widely used in tunnel crossing construction projects of Shenzhen and some projects in Shanghai.

**Fig. 7:**
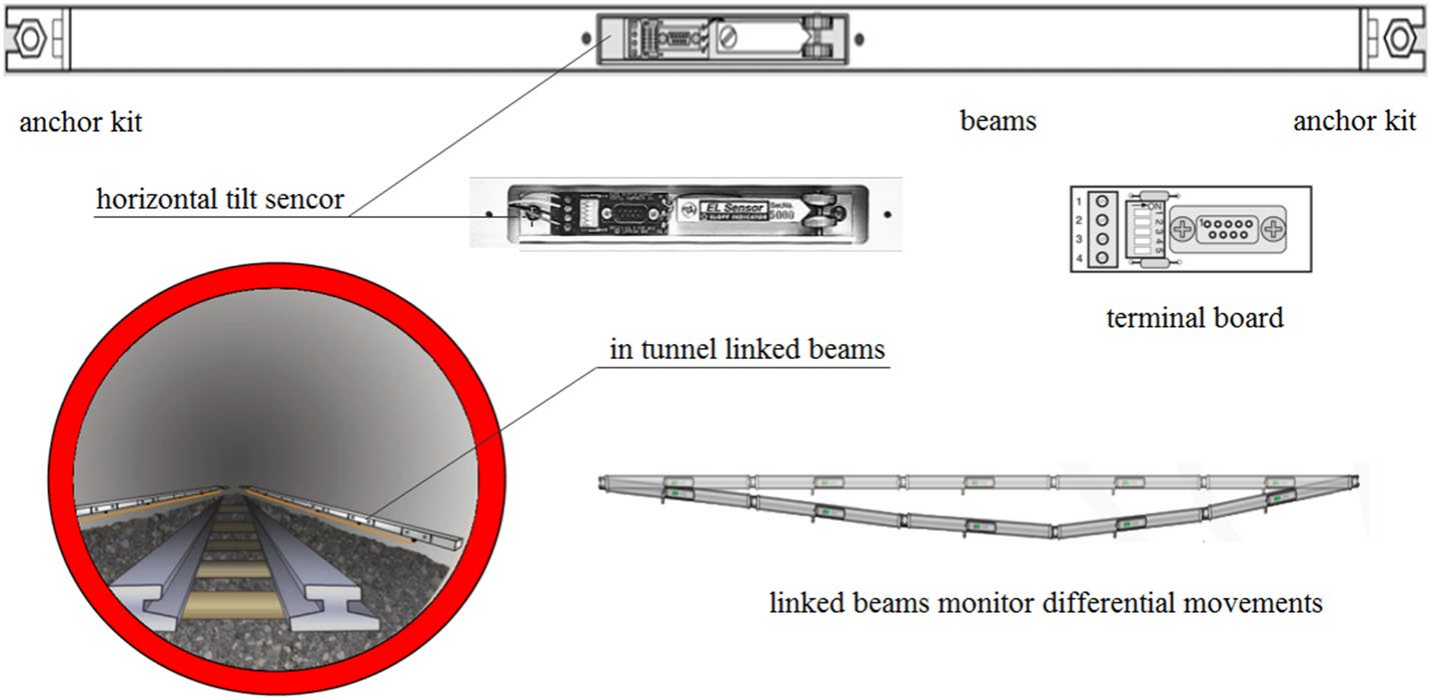
Electrolevel beam sensor monitoring system

Indicated in Fig. 7 is the electrolevel sensor, developed by Bassett et al. (1999), consisting of a rigid metal beam typically 1–2 m long with end brackets, two anchor kits set into the tunnel structure, an electrolytic tilt sensor (a precision bubble-level) sensed electrically as a resistance bridge whose circuit outputs a voltage proportional to the tilt of the sensor, and a terminal board. Linked electrolevel beam sensors can measure differential settlements and settlements of the tunnel structures within the influence zone. The electrolevel beam sensor measuring system was ever employed in almost all the tunnel crossing projects of Shanghai subway.

**Fig. 8:**
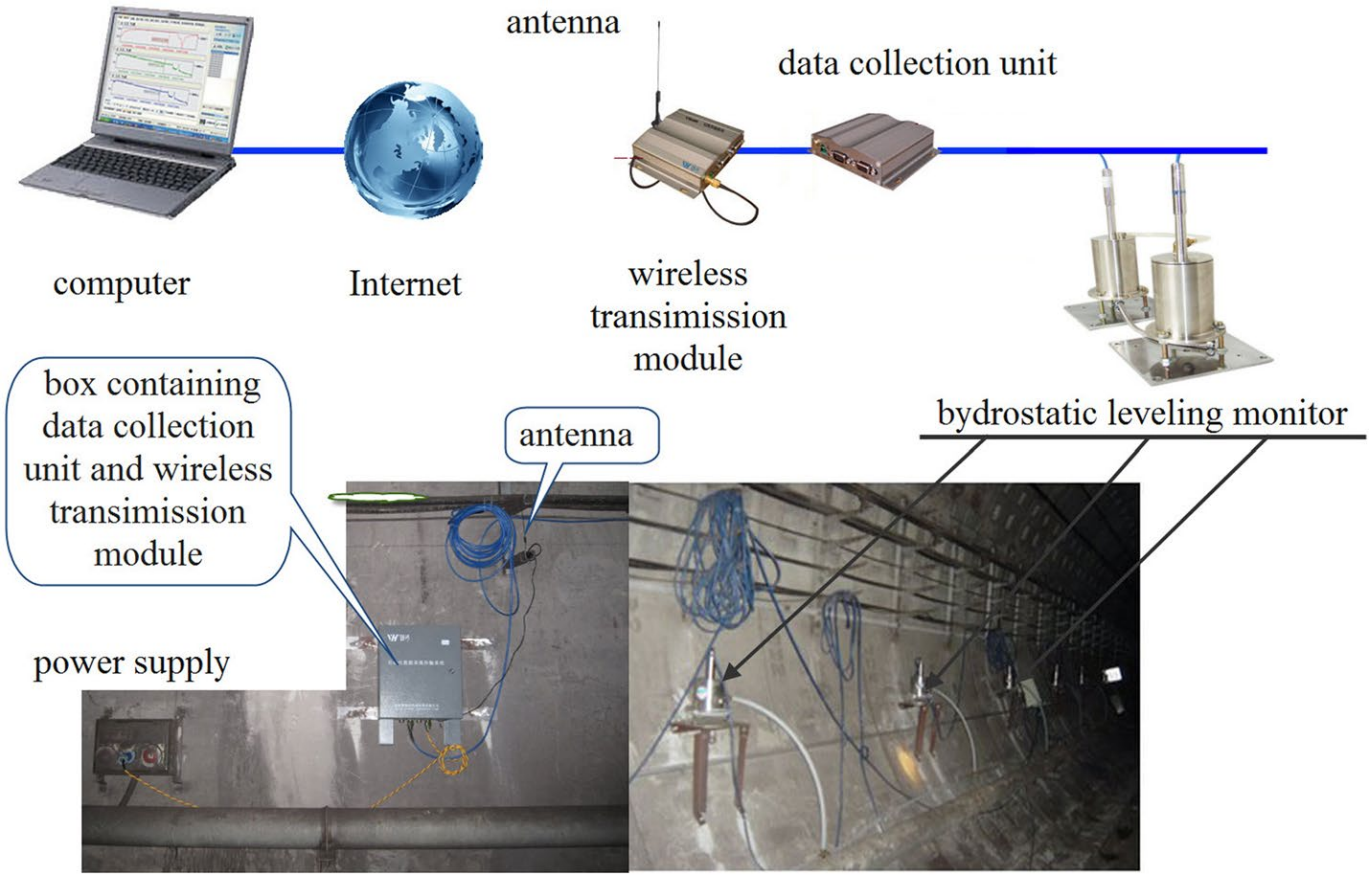
Hydrostatic leveling measuring system

Presented in Fig. 8 is the hydrostatic leveling system for measuring settlement profiles, consisting of a number of installed liquid filled pots, hydraulically connected to a reference pot located in a stable area. The elevation of the liquid in the reference pot is maintained constant by means of a minipump, reservoir and an overflow unit. LVDT float sensors monitor the height of the liquid in each pot. When settlement or heave occurs, the sensor detects the apparent change in the height of the liquid and transmits the signal to a data logger for continuous monitoring and real-time processing. This system is widely used in constructing tunnels in close proximity to the existing operational tunnels of Beijing subway.

**Fig. 9:**
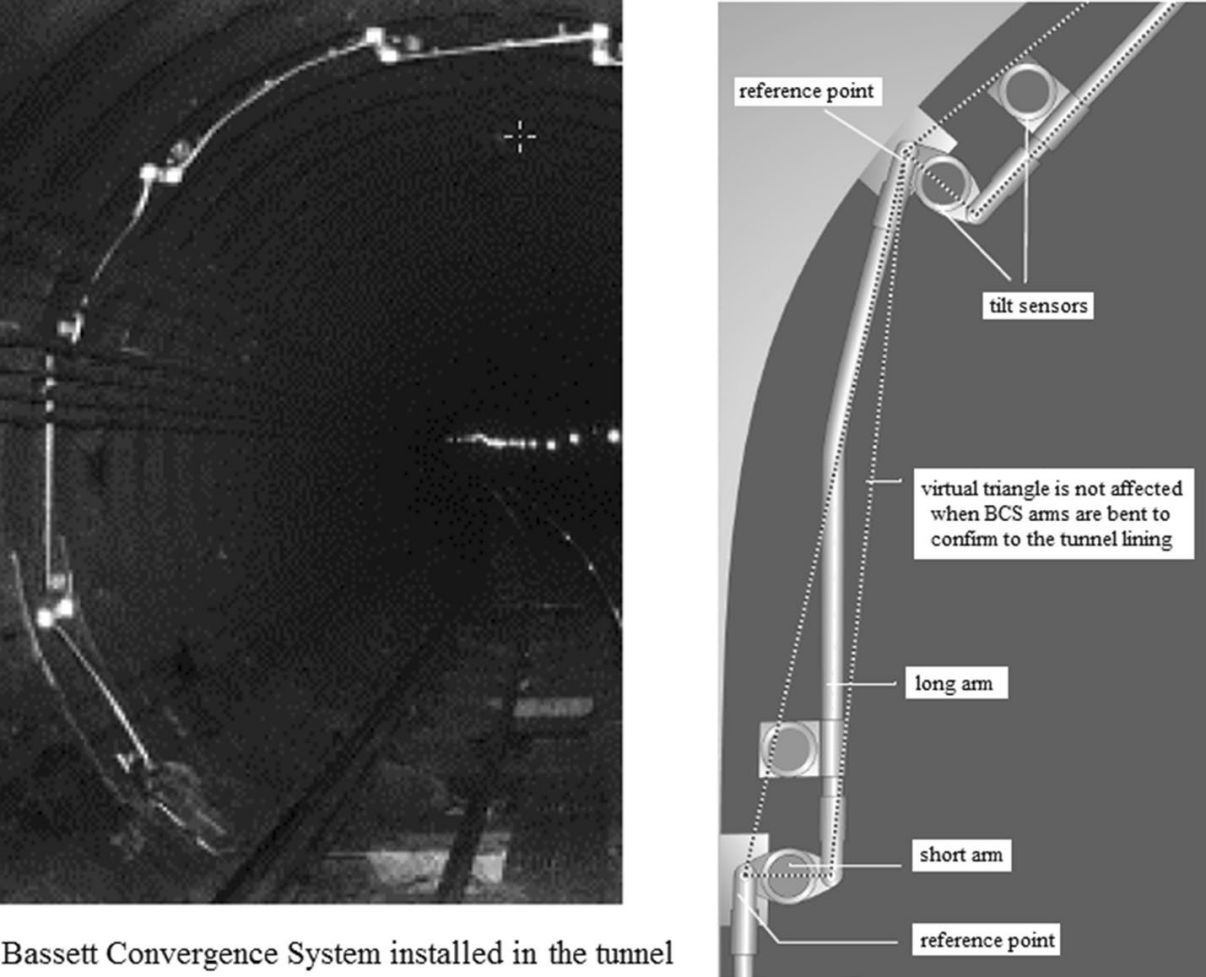
Bassett Convergence System used in Shanghai

Moreover, as shown in Fig. 9 the Bassett Convergence System (BCS) ever used in some tunnel of Shanghai subway to monitor the tunnel for potential damage from the nearby construction activities, consists of the articulated arms linking each reference point to the next and forming a series of virtual triangles. The BCS monitors movements of the reference points mounted on the tunnel lining. The reference points are aligned to a plane that is normal to the axis of the tunnel. A tilt sensor is mounted on each arm. Spatial displacement of the reference point moves the arms and results in changed tilt readings. Tilt readings are recorded by a data logger and retrieved at scheduled intervals by a remote computer.

**Fig. 10:**
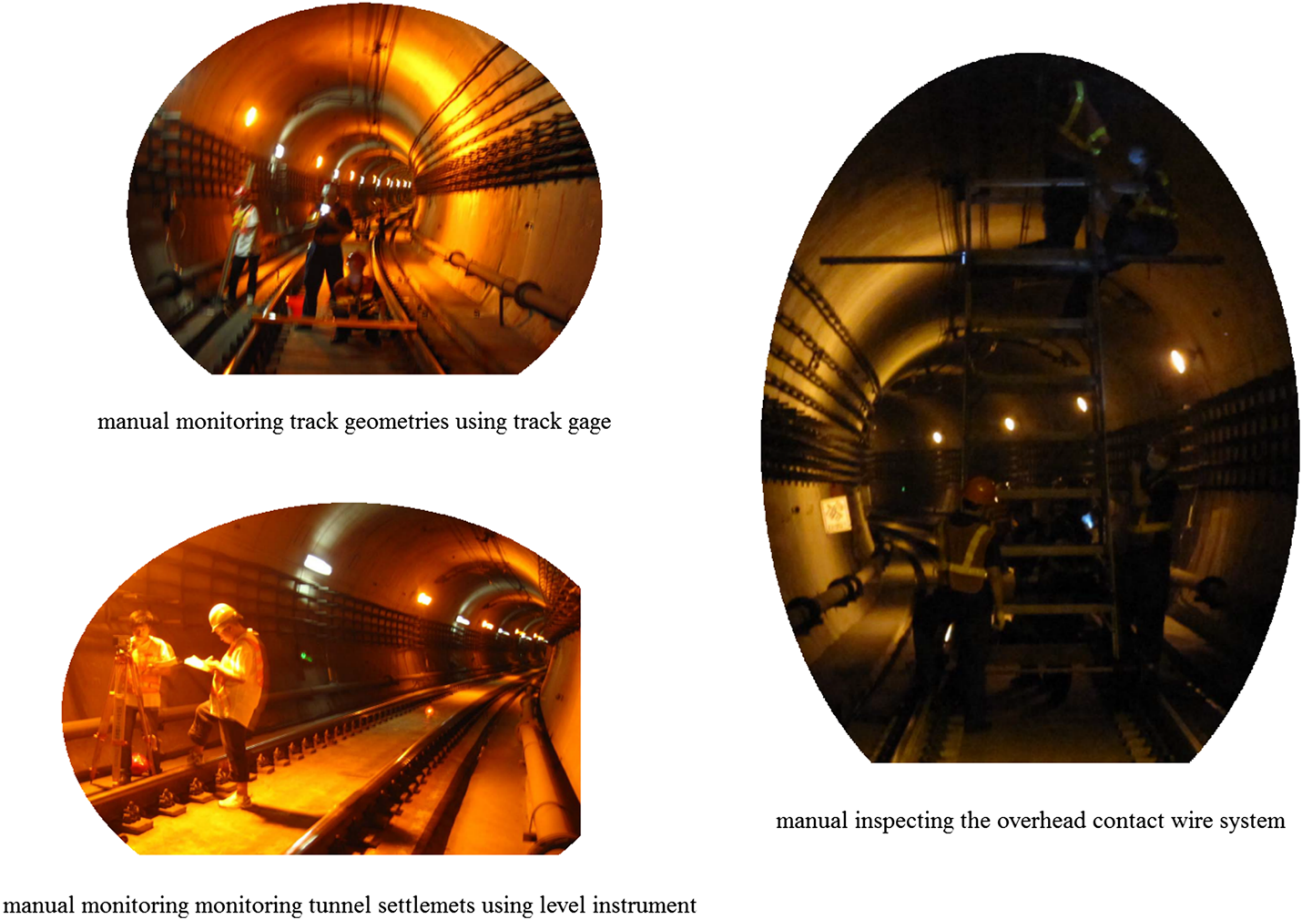
In-tunnel manual monitoring works

Considering that the in-tunnel environment particularly the train induced vibration will inevitably impact readings of the automatic monitoring systems, the manual method is indispensable to the monitoring plan (see Fig. 10). The remote reading method is supplemented, supervised and verified by the manual monitoring method.

Due to the reason of saving money, deformation of the track system is monitored by manual methods in most tunnel crossing construction projects of mainland China. After all, it is movement of the tunnel that result in the deformation of the track system. As to changes of the existing cracks in tunnel structures usually experienced engineers are assigned to inspect developments of the cracks at night. Other facilities, such as the overhead contact wire system, were often inspected manually at night and when the power supply was switched off, as shown in Fig. 10.

(2) Monitoring shield tunneling parameters

In order to reduce shield tunneling-induced movement of the existing tunnel to the largest degree, the construction should proceed as a controlled process. In the process, key shield performance on-board parameters need to be reviewed and updated constantly based on the measured data following each planned stretch of the new tunnel. So the subject of what to monitor is crucial to ensuring safety of the existing tunnel. The summary list of the monitoring aspect includes: (a) monitoring and adjustment of the face-support pressure, considering that the potential instability of the excavation face is the major source of risk or severe damage to the existing tunnels; (b) monitoring the state of the muck in light of samples of the excavated soils; (c) monitoring the excavation volume of each segmental ring; (d) monitoring volume and pressure of the simultaneous backfilling grouting; (e) monitoring advance rate of the shield machine; (f) monitoring rotation and torque of the cutter head as well as thrust of jacks. This work are carried out in usual shield tunneling and done with more persistence and endurance in tunnel close crossing construction.

(3) Threshold values for deformations of the tunnel and track

One of the central elements for design of the monitoring plan is to set threshold values for all the relevant indicators, which are deformations of the tunnel and track. To activate countermeasures and detect anomalous trends is performed in accordance with the determined threshold values.

Two threshold values, namely the ‘alarm’ and the ‘attention’ thresholds, are normally defined for deformations of the tunnels and tracks in China’s crossing projects. The attention threshold value, once reached, should help to draw the necessary attention of the Parties involved to the need for a more careful control of the shield tunneling process in order to stay below the alarm threshold. The alarm threshold value, once exceeded, would require that a decision be made on whether or not to activate immediately the corresponding predefined countermeasures. In practice, it is surely tough to decide the two threshold values. The two thresholds were usually decided for convenience of the risk management of the operational subway tunnels, and also a compromise of the involved parties. The alarm threshold for tunnel movement in shanghai was usually taken as 3 mm (Zhu and Huang 2010). In Shenzhen, the attention threshold and alarm threshold for tunnel movement are often taken as 50 and 80 % of the allowed movement respectively. In Beijing, the attention threshold and alarm threshold for tunnel movement are always taken as 70 and 80 % of the allowed movement respectively (Li 2010).

(4) Information management

One of the main objectives of the information management is to check and detect any anomalous trend in the monitored parameters. So the transfer and flow of measured data among the Parties involved is of vital importance to the tunnel close crossing construction projects and a collaborative-work environment can provide facilities for sharing data, tracing decisions, and communicating the information by efficient means. Usually a joint team consisting of the designer, the builder, the inspection engineer and the owner and the administrator of the existing operational tunnels were established and on duty in an office on ground surface of the subject construction site, constituting an indispensable tool for the efficient and effective construction management. In the office, discussion and analysis are ready to do according to monitored movements of the existing tunnels and parameters of the shield performance, to single out the safety condition of the operational subway tunnels, and decide whether, how, when and where to perform or adjust the predefined construction plan, and most important of all, to activate the corresponding countermeasures, taking in due consideration the timing factor, in accordance with predefined thresholds of the key parameters such as movements of the existing tunnels.

### Measures and activation mechanism of countermeasures

1. Measures and countermeasures

It is required to make a robust plan of countermeasures to be implemented in advance of tunneling, if thresholds of the key parameters are exceeded in mainland China. For extremely critical situations, an emergency plan is always to be prepared.

On account of the crowded ground surface in urban areas of China’s largest cities, the widely adopted measures including countermeasures mainly comprised limiting speed of trains, grouting work of shield tunneling and grouting work within existing subway tunnels. Limiting speed of the in-tunnel trains to 10–30 km/h was an important measure and widely accepted and employed. Sometimes adjustments of the train schedule are made on account of the reduction in speed of trains passing the influence zone on the existing tunnels. The grouting work of shield tunneling includes grouting of tail void (simultaneous backfilling grouting, first phase injection) and grouting directly through the segments (second phase injection) (Guglielmetti et al. 2008). The second phase injection was employed in case of movement of the existing tunnel demonstrates a trend of sharp increase, and it is subdivided according to the progress of a tunnel close crossing project and deformations of the operational subway tunnels (Li and Chen 2012).

Once the two-liquid cement-sodium silicate grout was widely used in shield tunneling grouting, especially the injection from segment holes (Shi 2006). The setting time of the grout can be adjusted, and its gel time may be accurately controlled within ranges of several minutes to dozens of hours. Early strength of the grout could be achieved as desired, as was very much helpful to reduce movement of the neighboring tunnel in water bearing sandy ground. Recently, a one-liquid type grout, used as the simultaneous backfilling material, consisting of sand, fly ash, bentonite, lime, water and water reducing agent, has been popularized in construction of the river-crossing tunnels and particularly the subway tunnels in Shanghai with an attempt to confine the long term settlement of the operational subway tunnels (Yang and Fan 2008; Huang and Zhang 2009; Chen and Li 2011; Xiao et al. 2011).

The adopted mix proportions of the first and the secondary phase injection are often predefined according to the test results in advance of tunneling or based on the gained construction experiences. For example, the injection volume of the first phase injection, suggested before crossing, should be changed in light of the shield driving and measured movements of the existing tunnels. The same is true of the parameters of the injection volume, location and frequencies of the secondary phase injection.

The grouting work within the existing tunnels is a particularly crucial countermeasure to control long term movements of the existing subway tunnels caused by new nearby shield tunneling in soft ground such as in Shanghai and Hangzhou, where the post-construction settlement of the existing tunnels can last several years. Adopting the two-liquid cement-sodium silicate grout, the scheme for controlling movements of the operational subway tunnels was formulated (Wang 2011; Feng et al. 2011; Xiao and Wang 2011).

(2) Activation mechanism of countermeasures

The countermeasures are those actions, which will be activated during construction according to predefined triggering criteria, should the key parameters reach predefined thresholds-attention and alarm thresholds. When attention thresholds are exceeded, a common countermeasure is to increase the frequency of the monitoring readings. This countermeasure help to detect if the alarm value is approached soon leading to overcoming the alarm thresholds. In parallel, a detailed review of the shield driving parameters is generally required. Countermeasures, aiming to reducing shield tunneling induced disturbances of the surrounding ground, often included:

- Adjusting the ranges of the shield driving parameters, particularly the advance rate and earth chamber pressure;
- Modifying parameters concerning conditioning agent for better pressurizing the excavated material in the excavation chamber;
- Executing additional probing ahead of the tunnel face and boreholes from the ground surface to check the geological and hydrogeological conditions if permitted;
- Carrying out more strict control of the simultaneous backfilling grouting;
- If necessary, employing the grouting through segment holes behind the shield machine.

When alarm values are reached and have been potentially exceeded, a full and thorough review must be performed of the design parameters based on monitoring and visual inspections of the shield performances such as state of the muck, and at the same time countermeasures, aiming at preventing the existing tunnel from deforming too much, will be implemented. Countermeasures always include:

- Grouting through segment holes once even more times in the new shield tunnel;
- Grouting in the existing tunnel especially when the tunnel close crossing construction project is completed and where the post-construction settlement of the tunnel takes the lead.

## Discussions

### Movement of the existing subway tunnel

Movement of the existing subway tunnel can be divided into the short-term movement, which occurs during construction of the new nearby shield tunnel, and the long-term movement (post-construction movement), which is tunnel settlement developed after the completion of the construction. The former is mainly decided by factors including the intersection angle and clear distance between the existing tunnel and the new tunnel, shield driving parameters, overburden depth and mechanical properties of surrounding soils and other adopted measures such as in-tunnel grouting; the latter, which is related to the primary consolidation and secondary compression of underlying soils, is governed by the time-dependent mechanical properties of the soils and can last several years even dozens of years. In cities like Beijing and Shenyang in China, for soil layers encountered in subway tunnel construction are chiefly sandy clays and sandy cobble layers, the post-construction tunnel settlement caused by solidification shrinkage of backfilling grout tend to be stable in a not very long time usually <1 year. While in cities like Shanghai and Hangzhou, subway tunnel are mostly constructed in water-bearing soft compressible cohesive soils and the post-construction tunnel settlement is far bigger than that accumulated in construction. Accordingly, the two types of tunnel movements necessitate separate discussion.

1. Operational tunnel movement in construction

**Fig. 11:**
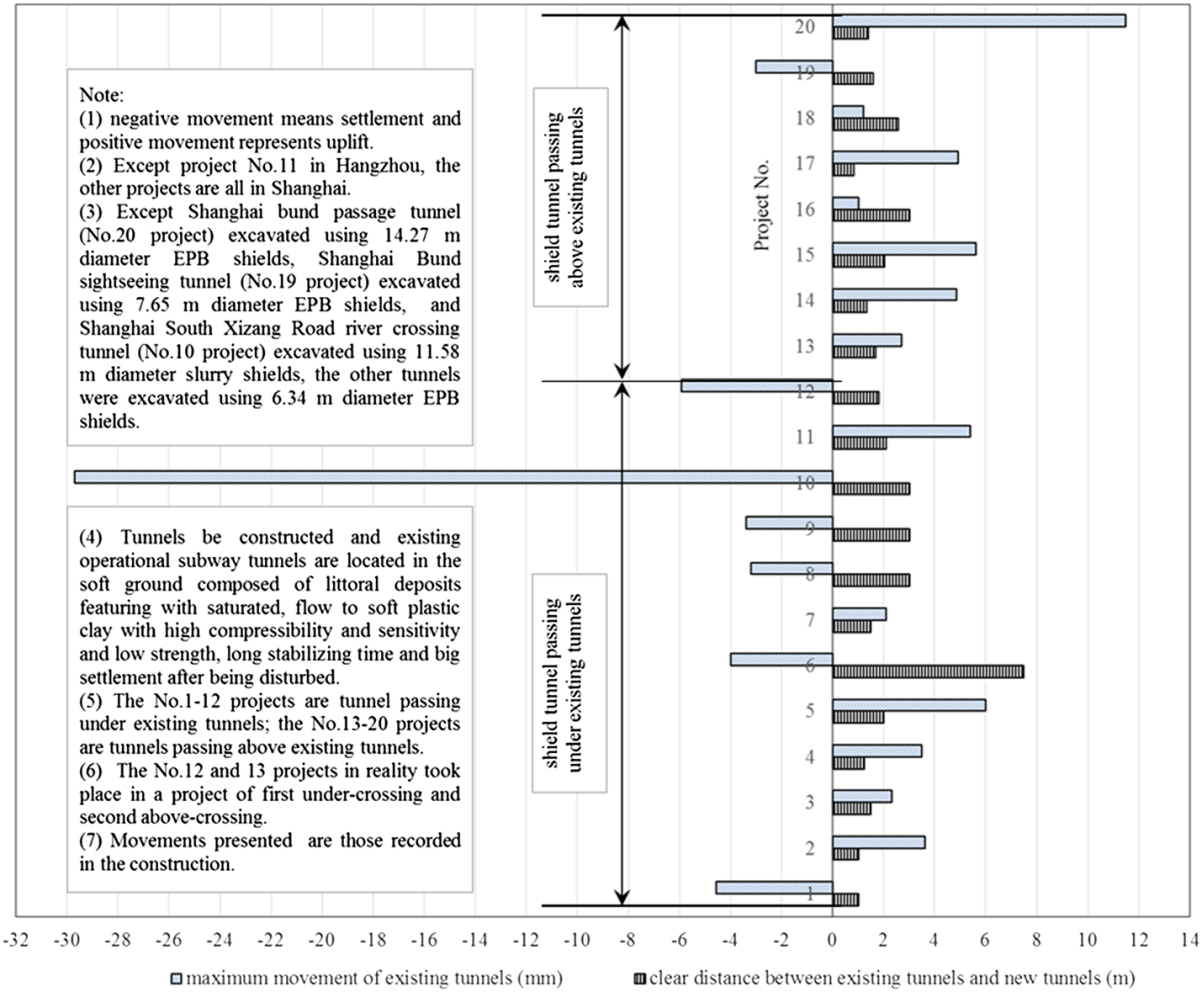
Maximum vertical movements of the existing tunnels in Shanghai and Hangzhou

The clear distance between new lines and existing lines is a crucial parameter in planning subway line systems, which determines the longitudinal slope and alignment of the new lines. Generally, the shorter the clear distance of a tunnel crossing construction project, the higher the longitudinal slope of the new tunnels. A shorter clear distance is usually a better choice. However, the shorter clear distance between new tunnels and existing tunnels means the higher construction risk over the whole period of the tunnel crossing construction. Investigation of the existing tunnel movement under different clear distances is of great significance. The maximum movements of the existing tunnels are given in Fig. 11 together with the clear distances of 20 projects (19 projects in Shanghai and one in Hangzhou). The background of each project is briefly presented in Table 4.

**Table 4:** Cases of shield tunneling adjacent to operational subway tunnels in Shanghai and Hangzhou

Project no.	Tunnels be constructed	Tunnel crossing	Existing tunnels	Source of citation
1	Shanghai subway Line 2 tunnels	Approximate vertical under-crossing	Subway tunnels on Line 1	Bai et al. (2000)
2	Shanghai subway Line 4 tunnels	Under-crossing along a 380 m radius curve	Subway tunnels on Line 2	Wang et al. (2004)
3	Shanghai subway Line 7 tunnels	Under-crossing at a skew of 98°	Subway tunnels on Line 1	Chen et al. (2011)
4	Shanghai subway Line 7 tunnels	Under-crossing at a skew of 40°	Subway tunnels on Line 2	Zhu and Huang (2010)
5	Shanghai subway Line 7 tunnels	Under-crossing	Subway tunnels on Line 2	Zhu and Huang (2010)
6	Shanghai subway Line 11 tunnels	Under-crossing at a skew of 30°	Subway tunnels on Line 1	Pan (2011)
7	Shanghai subway Line 11 tunnels	Under-crossing	Subway tunnels on Line 2	Wang (2011)
8	Shanghai EXPO power able tunnel	Under-crossing at a skew of 86°	Subway tunnels on Line 2	Yan et al. (2009)
9	Shanghai EXPO power able tunnel	Under-crossing	Subway tunnels on Line 4	Han (2013)
10	Shanghai South Xizang Road river crossing tunnel	Under-crossing at a skew of 56°	Subway tunnels on Line 8	Jiang (2009)
11	Hangzhou subway Line 4 twin running tunnels	Under-crossing	Up-line tunnel of Line 1	Not reported
12	Shanghai subway Line 11 up-line tunnel	Under-crossing at a skew of 75°	Subway tunnels on Line 4	Wang et al. (2014)
13	Shanghai subway Line 11 down-line tunnel	Above-crossing at a skew of 75°		
14	Shanghai subway Line 8 tunnels	Above-crossing at a skew of about 75°	Subway tunnels on Line 2	Wang and Yu (2006)
15	Shanghai subway Line 10 tunnels	Above-crossing	Subway tunnels on Line 1	Zhu and Huang (2010)
16	Shanghai subway Line 13 tunnels	Above-crossing at a skew of about 76°	Subway tunnels on Line 4	Zhu and Huang (2010)
17	Shanghai subway Line 9 tunnels	Above-crossing	Subway tunnels on Line 1	Zhu and Huang (2010)
18	Shanghai subway Line 9 in-and-out section tunnel	Above-crossing at a skew of 18°	Main line tunnel of Line 9	Chen et al. (2009)
19	Shanghai Bund sightseeing tunnel	Above-crossing at a skew of about 51°	Subway tunnels on Line 2	Zhou and Wu (2000a, b)
20	Shanghai Bund passage tunnel	Above-crossing at a skew of 75°	Subway tunnels on Line 2	Yuan (2012)

Usually existing tunnels settle in under-crossing construction and uplift in above-crossing construction. But in Fig. 11 the tunnel movements of 6 out of 12 under-crossing projects are minus movements, which represent uplift. The phenomena can be attributed to the higher face pressure adopted in the construction and the sensitive soft clay in Shanghai and Hangzhou, which can effectively transmit the pressure onto the existing tunnels, causing uplift of the existing tunnels. The existing tunnel in the No. 19 above-crossing project settled, for the higher grouting pressure in the new tunnels was used to improve the overlying soil of the existing tunnels. In short, it is in the sensitive soft clay of Shanghai and Hangzhou that using face supporting pressure and grouting pressure to actively control movements of the existing tunnels can be realized, especially in case of thinner soil layers between new tunnels and existing tunnels. From Fig. 11, the movements of the existing tunnels are in the ranges of −6 to +8 mm except existing tunnels of the Nos. 10 and 20 projects having larger movements of −29.68 and 11.46 mm respectively, and these two larger movements can be attributed to excavation diameter more than 10 m of the used shield machines.

**Fig. 12:**
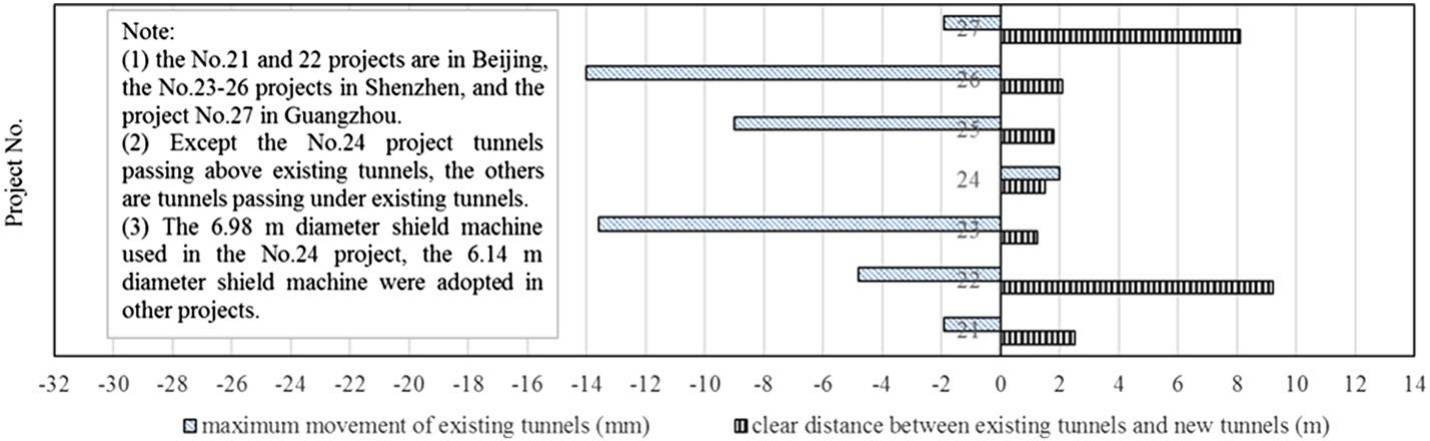
Maximum vertical movements of existing tunnels of shield tunnel crossing projects in Beijing, Shenzhen and Guangzhou

The collected construction cases in Beijing, Shenzhen and Guangzhou are displayed in Fig. 12 and Table 5. Evidently, the existing tunnels follow the usual rule of settling in under-crossing and uplifting in above-crossing. Not including the No. 23 project crossing in the launching phase and the No. 26 project crossing along a 350 m radius curve, the measured maximum tunnel movements of other projects are <10 mm. Difficult shield driving control resulted in the larger settlements of tunnels in the two projects. For presented cases herein, the tunnels are mainly buried in the layers of weathered granite, gravely clay and sandy clay. Theses soils are not sensitive to the supporting pressure and grouting pressure compared with the soft clay encountered in Shanghai and Hangzhou, and shield driving and grouting measures play the role of confining movements of existing tunnels. On the whole, it is observed from Figs. 11 and 12 that the movement of the existing tunnel can be controlled within 15 mm even if the clear distance is only 1 m and when excavation diameters are <7 m of shield machines.

**Table 5:** Cases of shield tunneling adjacent to operational subway tunnels in Beijing, Shenzhen and Guangzhou

Project no.	Tunnels be constructed	Tunnel crossing type	Existing tunnels	Source of citation
21	Subway tunnels on Line 8 of Beijing subway	Under-crossing	Guloudajie station of Line 2	Jiang (2013)
22	Subway tunnels on Line 10 of Beijing subway	Under-crossing	Shaoyaoju station of Line 13	Yang et al. (2009)
23	Subway tunnels on Line 3 of Shenzhen subway	Under-crossing in launching phase	Subway tunnels on Line 1 of Shenzhen subway	Fan (2014)
24	Line 11 running tunnel of Shenzhen subway	Above-crossing	Line 1 running tunnels	Li et al. (2014)
25	Line 2 tunnels of Shenzhen subway	Under-crossing at a skew of about 55°	Line 1 tunnels	Li and Yuan (2012)
26	Line 2 tunnels of Shenzhen subway	Under-crossing at a skew of about 20°–23° and along a 350 m radius curve	Line 1 tunnels	Li and Chen (2012)
27	Line 6 running tunnel of Guangzhou subway along a 250 m radius curve	Under-crossing	Huangsha station of Line 1	Wang et al. (2011)

(2) Post-construction tunnel movement

Study on post-construction tunnel settlement is rather limited. A typical example of continuous tunnel settlement after the completion of construction was presented by Cooper et al. (2002), who studied the performance of the existing cast iron–lined tunnel on Piccadilly Line due to tunneling. The Piccadilly Line tunnel settled another 12 mm within 38 months after the completion of the crossing tunnel below. Another relevant piece of work is that of Ng et al. (2013). They investigated the mechanisms of long-term tunnel settlement Shanghai Subway Line 1 based on the measured long-term tunnel settlement of Shanghai subway Line 1 from 1994 to 2007, which continued with time and reached a maximum of 288 mm. Four possible causes-namely, effects of tunnel construction, cyclic loading due to running trains, secondary compression of soft clay, and groundwater pumping in sandy aquifers-were analyzed. Here discussed is the tunnel crossing construction caused long-term settlement of the existing tunnel. Xu et al. (2002) concluded that the ground surface settlement after construction is in direct proportion to that in construction based on in situ measured results when constructing the Shanghai Bund sightseeing tunnel using a 7.65 m diameter EPB shield machine. In Shanghai very soft clayey ground, consolidation settlement after construction is about a factor of 2–2.5 the settlement in construction, and it can develop at least half a year or more to become stable (Liao et al. 2009). Tunnel uplift after the construction deserves special attention. It was reported by Chen et al. (2006) that the most dangerous period for an operational subway tunnel above-crossed in Shanghai by a new shield tunnel is within 10–15 days after completing the tunnel close crossing construction, and the uplift of the existing tunnel in this period accounts for 70 % of the final uplift.

### Remedial grouting

The second phase injection adopted to reduce and mitigate the long term settlement of existing tunnel after construction are based on measured data of the tunnel settlement. For example, a combination methodology of the “observational method” and the “predefined design method” to deal with parameters concerning the grouting control of shield tunneling were discussed through a Case Study in Shenzhen Subway Construction (Li and Chen 2012). In Shanghai, the grouting principles for the second phase injection were concluded as grouting uniformly at small volume and more points for more times, and the grouting parameters such as the number of grouting holes and their positions, grouting length and pulling speed as well as the grouting pressure and the grouts flow rate, were pre-selected based on the above principles before the final execution, but still needed to be optimized in the process of grouting according to the feedback data of displacement (Liao et al. 2009; Wang et al. 2009). In some tunnel close construction projects of Shanghai, grouting as one of the main remedial measures had been used to rectify excessive settlement of the operational subway tunnel. Applications of the remedial grouting in two cases are introduced below.

1. Case 1: Shanghai subway Line 11 tunnels crossing under Line 2 tunnels (Wang 2011)

**Fig. 13:**
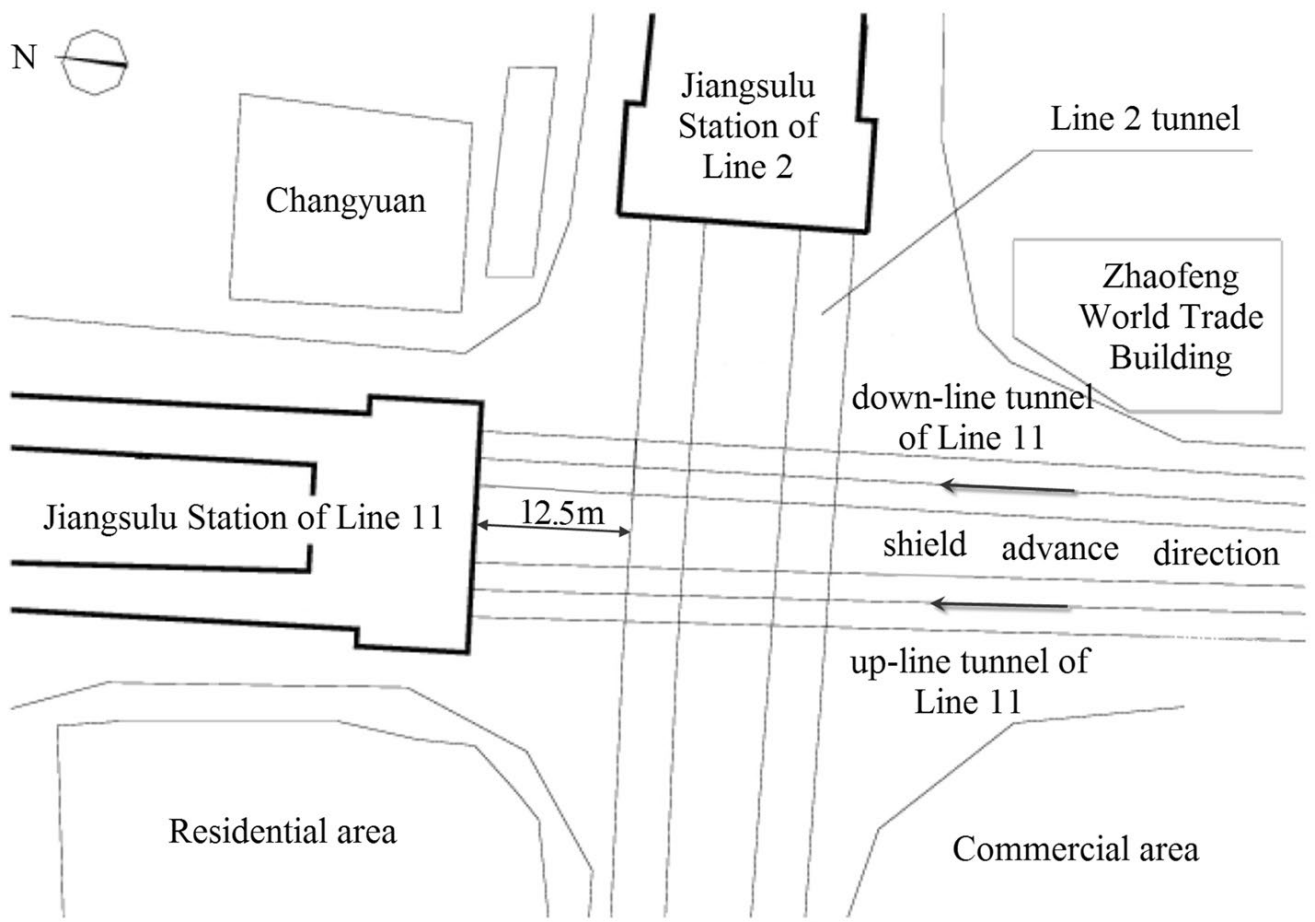
Plan view of Line 11 tunnels crossing under Line 2 tunnels

**Fig. 14:**
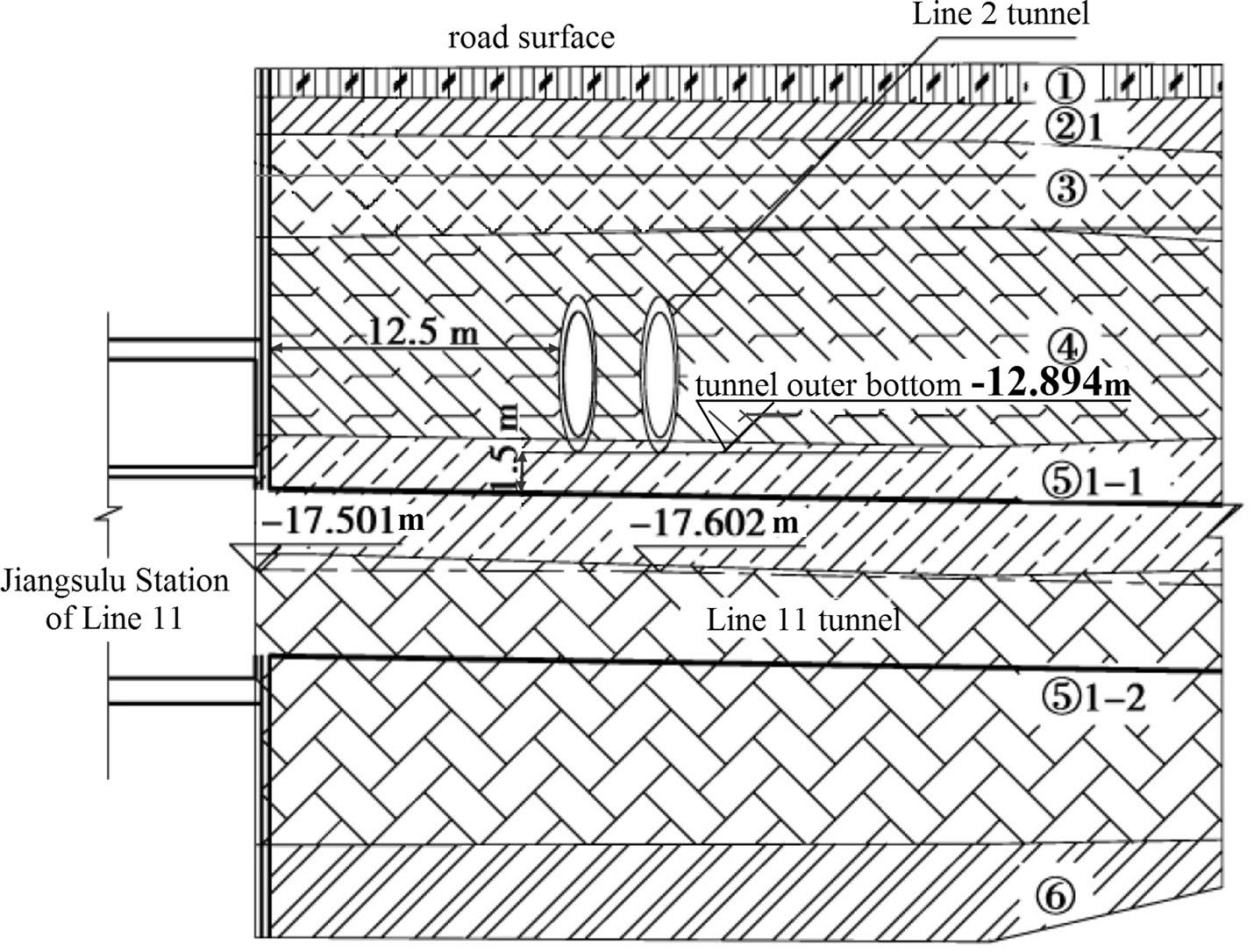
Cross section of Line 11 tunnels crossing under Line 2 tunnels

**Fig. 15:**
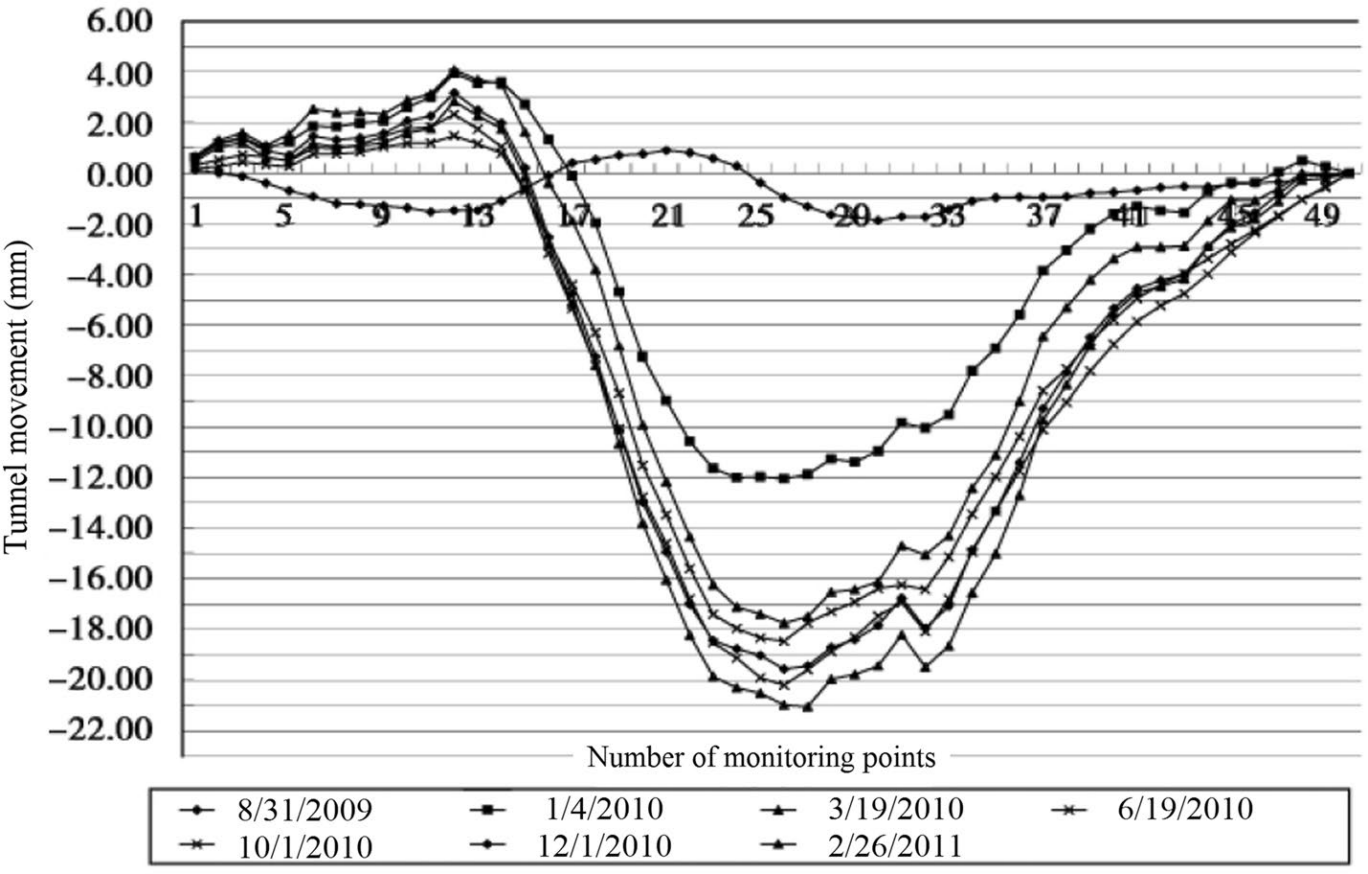
Movement of Line 2 down-line tunnel

**Fig. 16:**
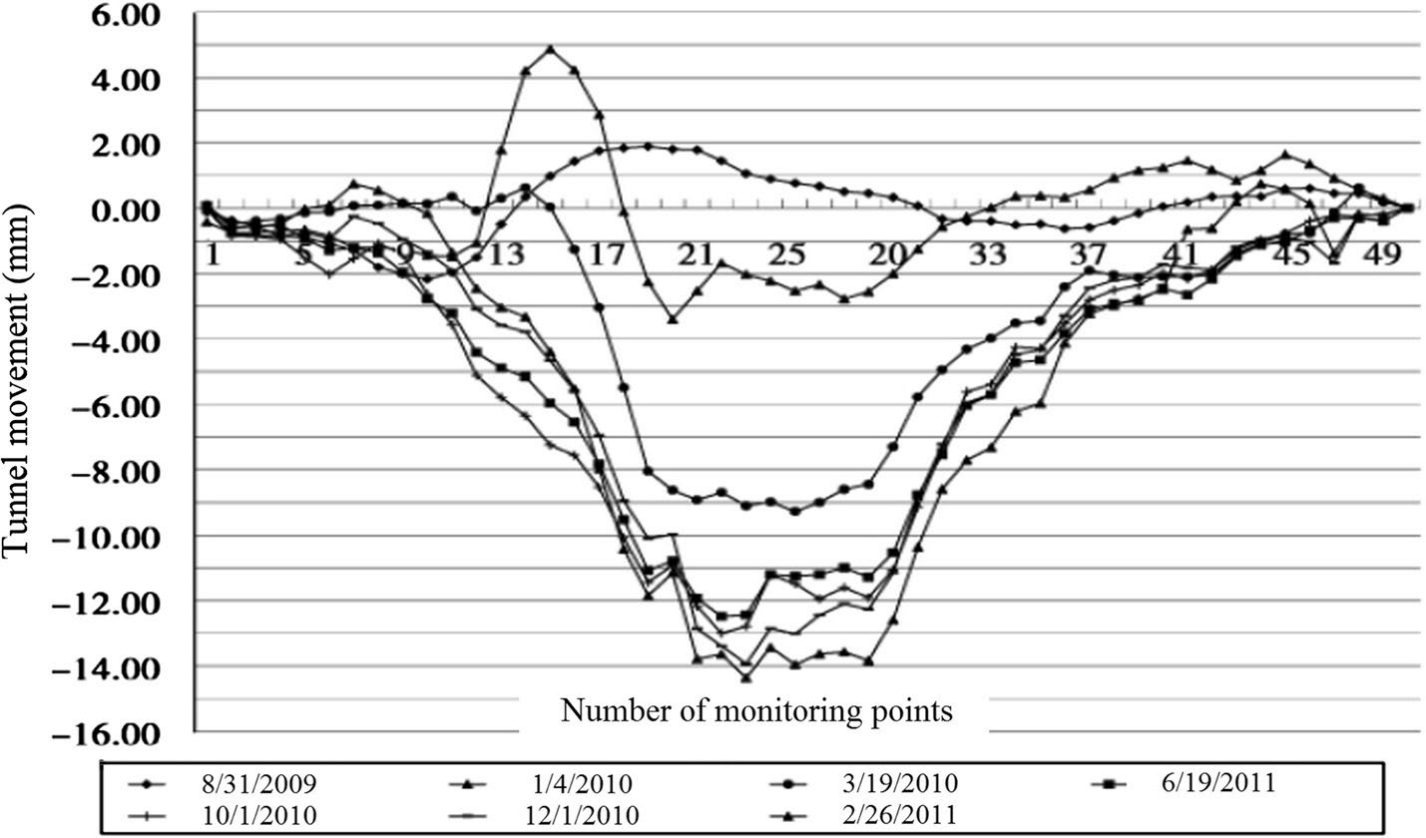
Movement of Line 2 up-line tunnel

As shown in Figs. 13 and 14, Shanghai subway Line 11 tunnels pass 1.5 m under the existing Line 2 tunnels. The ground at the construction site consists of backfill ➀, silty clay ➁_1_, muddy silty clay ➂, muddy clay ➃, clay ➄_1–1_, silty clay ➄_1–2_ and silty clay with clay silt interbed ➅. The existing tunnels are mainly buried in the muddy clay ➃, which is prone to developing long-term settlement. Constructing Line 11 tunnels underneath Line 2 tunnels was accomplished in August 2009, and the maximum settlements of Line 2 down-line and up-line tunnels were both <2.0 mm, which was well below 5 mm of the established tolerance. But the settlements of Line 2 tunnels continued to rise after the construction. As presented in Figs. 15 and 16, the maximum settlements of Line 2 down-line and up-line tunnels in February 2011 reached about 21.0 and 15.0 mm respectively. This larger settlements of Line 2 tunnels posed serious threat to safety of the above subway tunnel operation. So a remedial program of using grouting in Line 2 tunnels to reduce the excessive settlements was employed after subway service was halted at night.

**Fig. 17:**
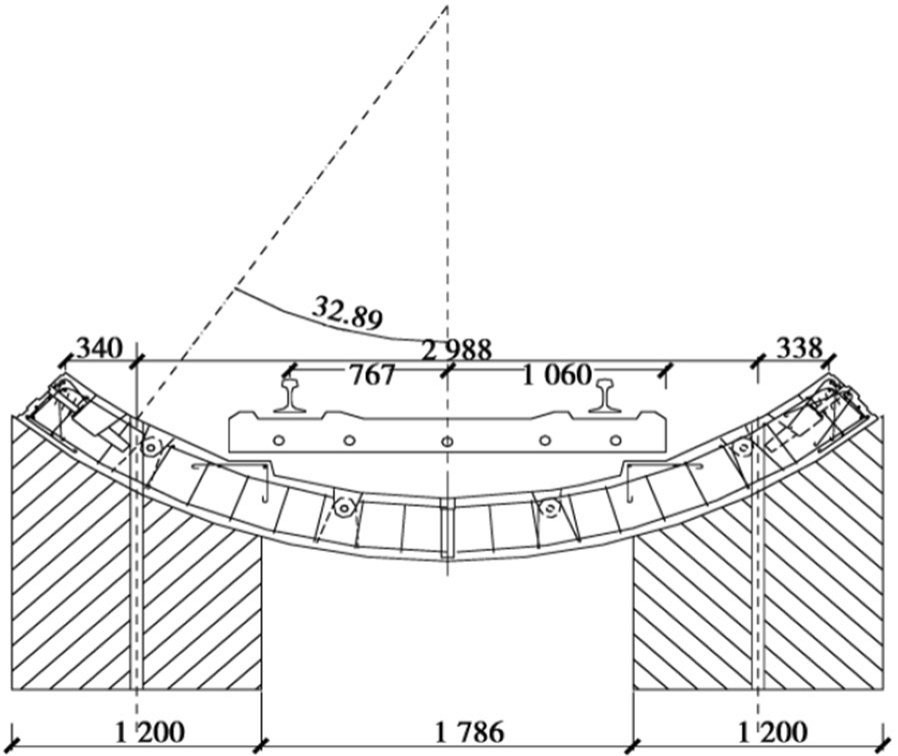
Positions of two drilled grout holes (unit: mm)

In the program, two grout holes were drilled vertically at the bottom of each segmental ring, as presented in Fig. 17. The grouting principles were concluded as grouting uniformly at small volume and more points for more times. Approximately 40 segmental rings within the settlement range of each tunnel was grouted, The two-liquid type grout of cement-sodium silicate with volume ratio 3:1 was adopted, and water to cement ratio was taken as 0.6–0.7. A steady flow of grout was ensured, and the flow rate was 20 L/min (14–16 L/min of cement and 4–6 L/min of sodium silicate).

To uniformly raise the settled tunnels, staggered grouting through one out of three successive spaced holes was executed and repeated, and the time interval for grouting in a hole was usually not <48 h, and the once grouting height and volume in the retraction grouting were controlled within 20 cm and 80 L respectively and adjusted according to the monitored tunnel settlement and observed grouting effects. The duration time for once grouting was about 4 min. Retraction rate of the injection tubes was decided by the flow rate of the two-liquid type grout, the once injection volume and injection height, and could be expressed as

1 $$v = l /\left( {V /q} \right)$$

where *v* is retraction rate of the injection tube, cm/min; *l* is once injection height, cm; *V* is once injection volume, L; and *q* is the flow rate of the two-liquid type grout, L/min.

For one hole, grouting two to three times was executed weekly. The maximum injection depth was 25 cm from Line 11 tunnel lining. The remedial grouting was carried out every day in the 3 h after subway trains stopped, and this work lasted more than 3 months.

**Fig. 18:**
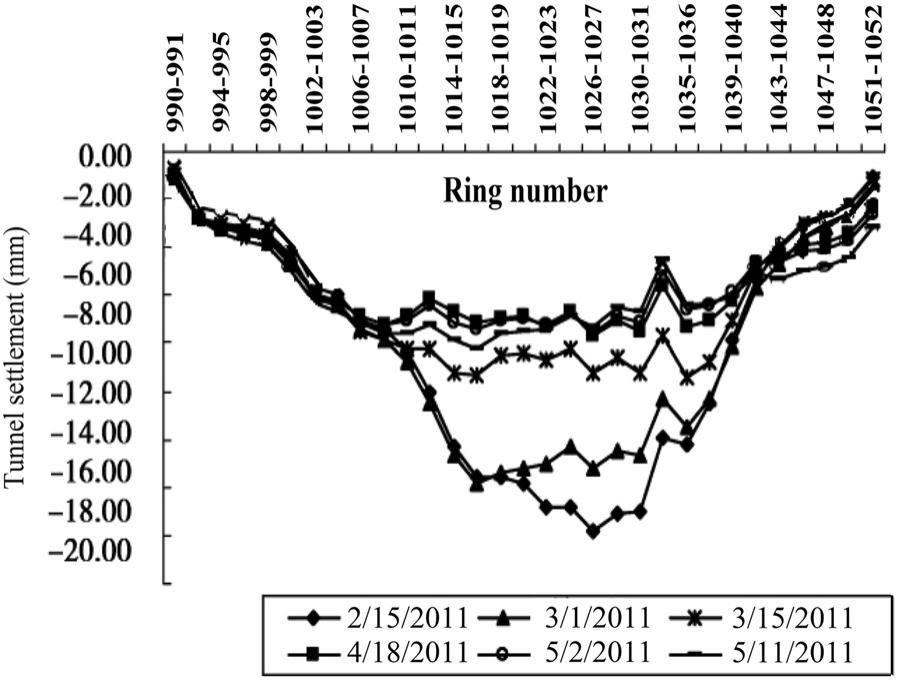
Settlement profile of Line 2 up-line tunnel

**Fig. 19:**
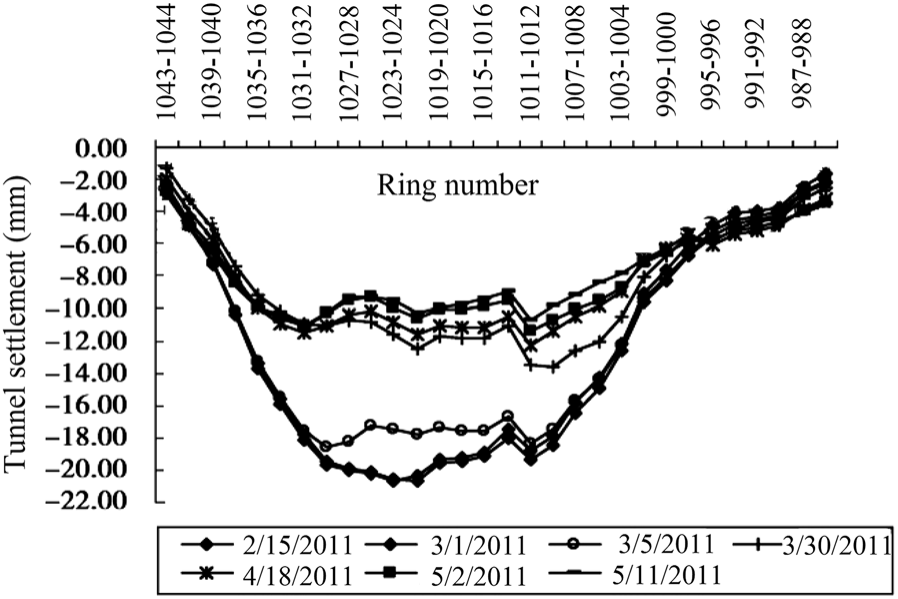
Settlement profile of Line 2 up-line tunnel

As given in Figs. 18 and 19, Line 2 up-line and down-line tunnels on the whole were lifted about 8 and 11 mm respectively after finishing the remedial grouting; the settlements of the two tunnels were controlled within 7 and 10 mm. The settlement profiles of Line 2 tunnels were well improved and guaranteed safety of the subway operation.

**Fig. 20:**
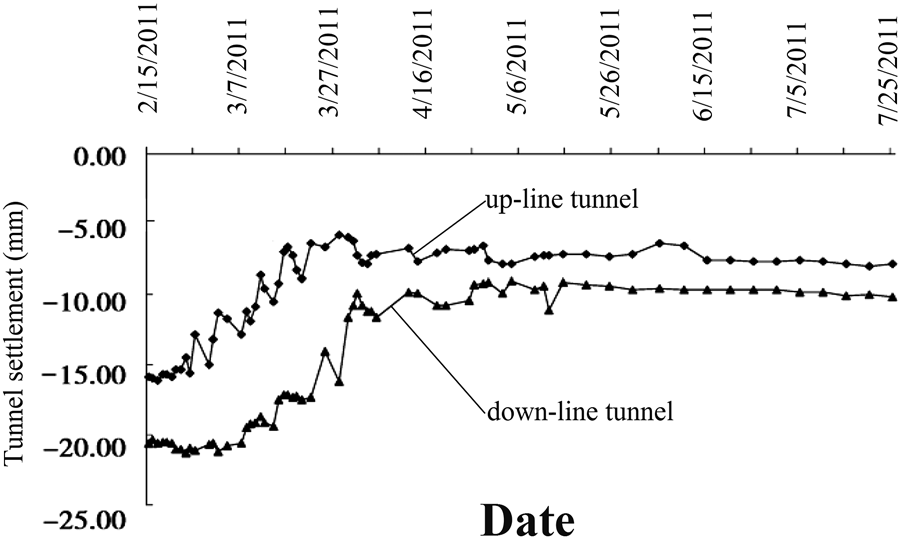
Settlements of Line 2 tunnels during and after the grouting construction

Also displayed in Fig. 20 are the settlements of Line 2 tunnels at the maximum settlement locations during and after the grouting construction. It is found that the remedial grouting substantially reduced the tunnel settlements at the beginning with the increase in injection depth and volume, but it was no longer effective when a certain amount of tunnel uplift was achieved of about 10 mm.

**Fig. 21:**
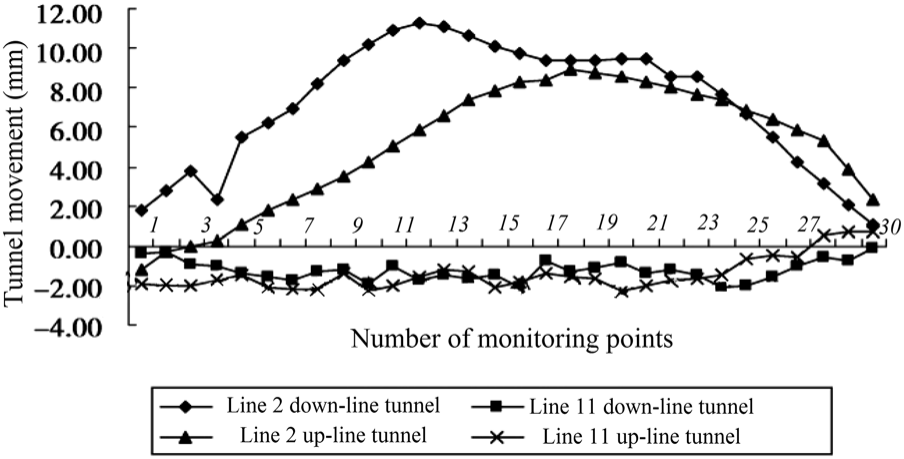
Monitored tunnel movements at the intersections of Line 2 and 11 tunnels

Being the clear distance of only 1.5 m between Line 2 and Line 11 tunnels, the grouting work inevitably induced settlements of the below Line 11 tunnels. Monitored tunnel movements at the intersections of the tunnels are listed in Fig. 21, which shows that the maximum settlement of Line 11 tunnels is about one-fifth of the maximum uplift of Line 2 tunnels.

**Fig. 22:**
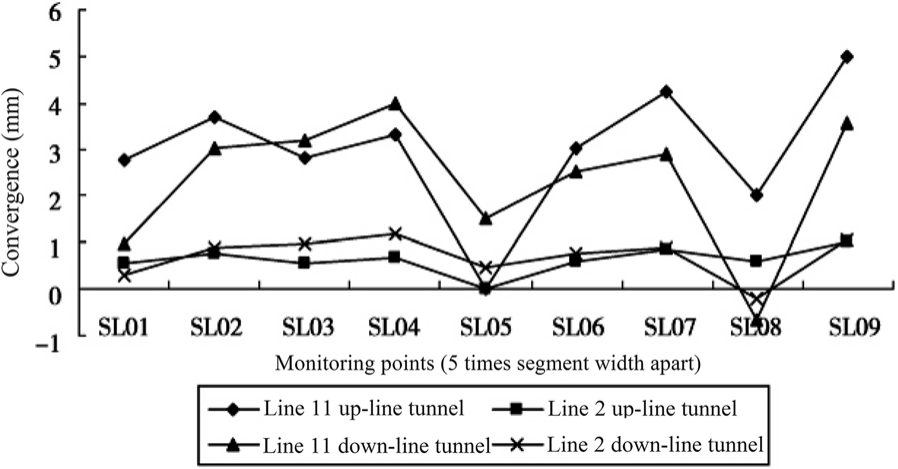
Convergences of Line 2 and 11 tunnels

Also recorded in Fig. 22 was the increase in tunnel diameter in horizontal direction (convergence) of the involved tunnels. The maximum convergence of the below tunnels was approached to 5 mm, while most convergences of the above tunnels <1.0 mm. Obviously, the grouting work in Line 2 tunnels produced more convergences of Line 11 tunnels. Therefore, adverse influences such as settlements and convergences on the below tunnels must be carefully monitored and managed when using in-tunnel grouting to lift and restore the above settled tunnels. Particularly, more attention must be paid to the tunnel convergences.

(2) Case 2: South Xizang Road River Crossing tunnels passing under Line 8 tunnels (Yang and Fan 2008)

**Fig. 23:**
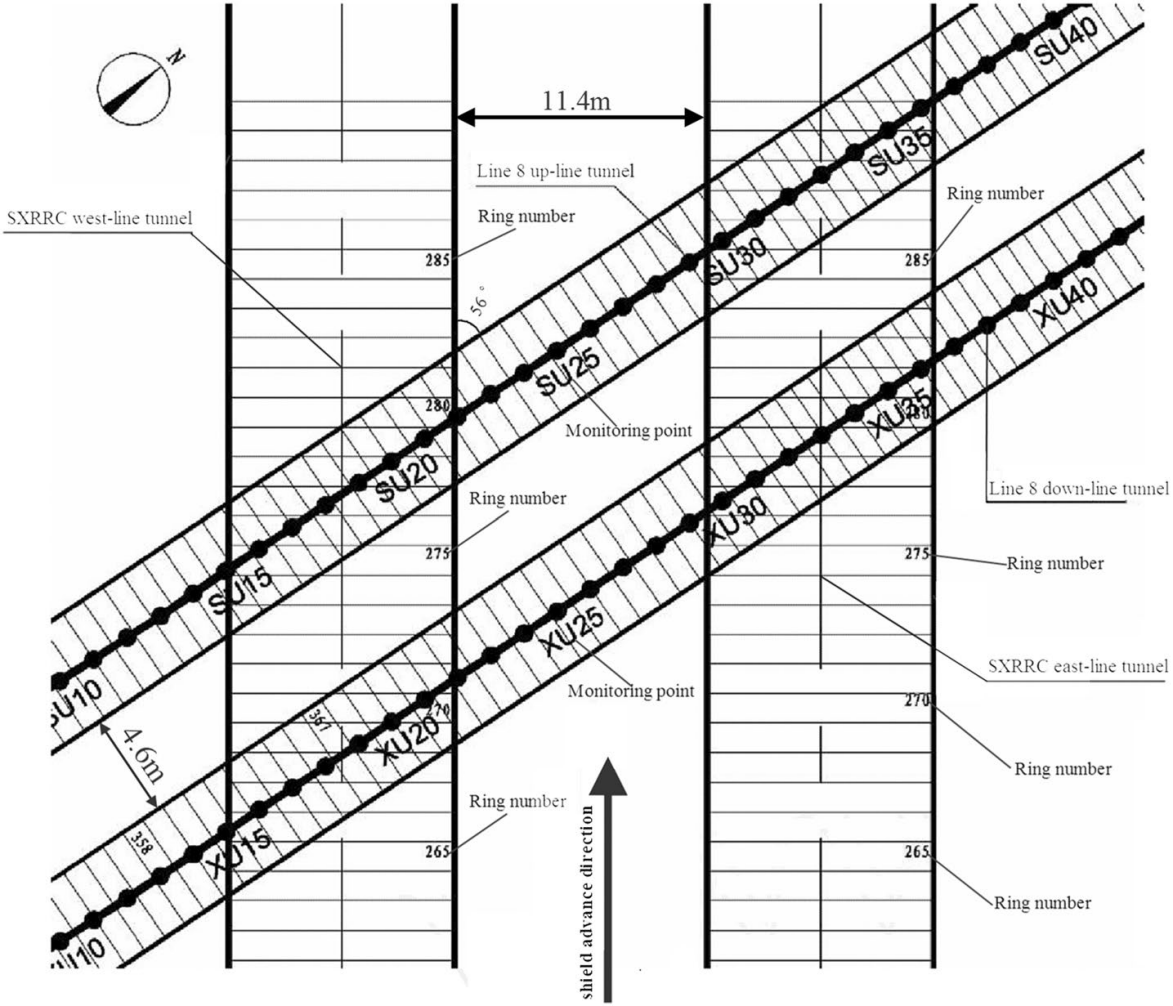
Plane of layout of the SXRRC tunnels and Line 8 tunnels

**Fig. 24:**
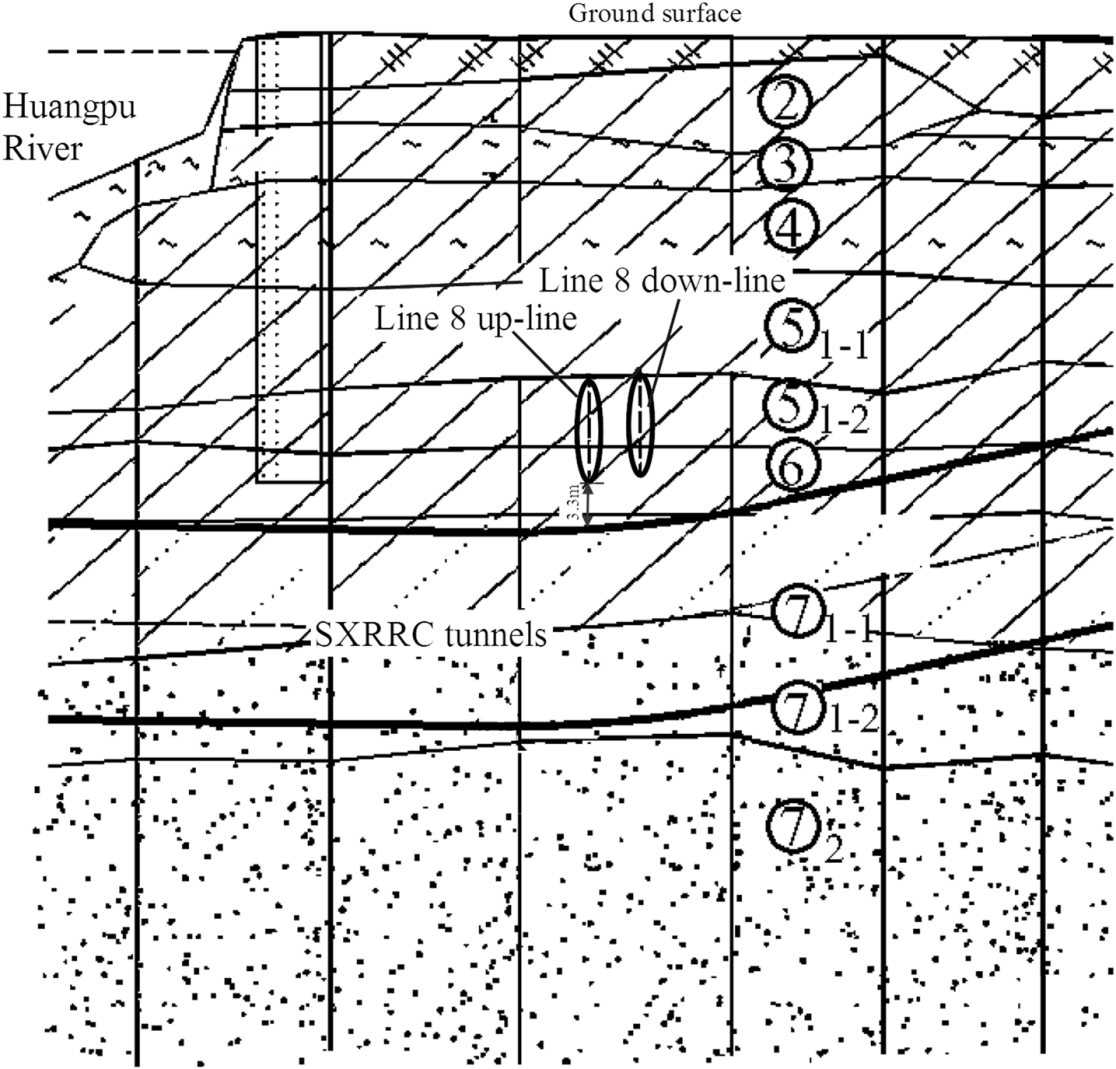
Cross-section of the SXRRC tunnels and Line 8 tunnels

The South Xizang Road River Crossing (SXRRC) tunnels obliquely cross under Line 8 tunnels at an angle of 56°, as shown in Figs. 23 and 24. The clear distance between the SXRRC tunnels and Line 8 tunnels is 3.3 m. The SXRRC west-line and east-line tunnels were constructed by two 11.58 m-diameter slurry shields and Line 8 tunnels constructed by two 6.34 m-diameter EPB shields. The ground at the subject construction site mainly consists of backfill ➀, silty clay ➁_1_, muddy silty clay ➂, muddy clay ➃, grey clay ➄_1–1_, grey sandy clay ➄_1–2_, dark green silty clay ➅, straw yellow sandy clay ➆_1–1_, grey yellow clayey sand ➆_1–2_, and grey yellow to grey silty-fine sand ➆_2_.

**Fig. 25:**
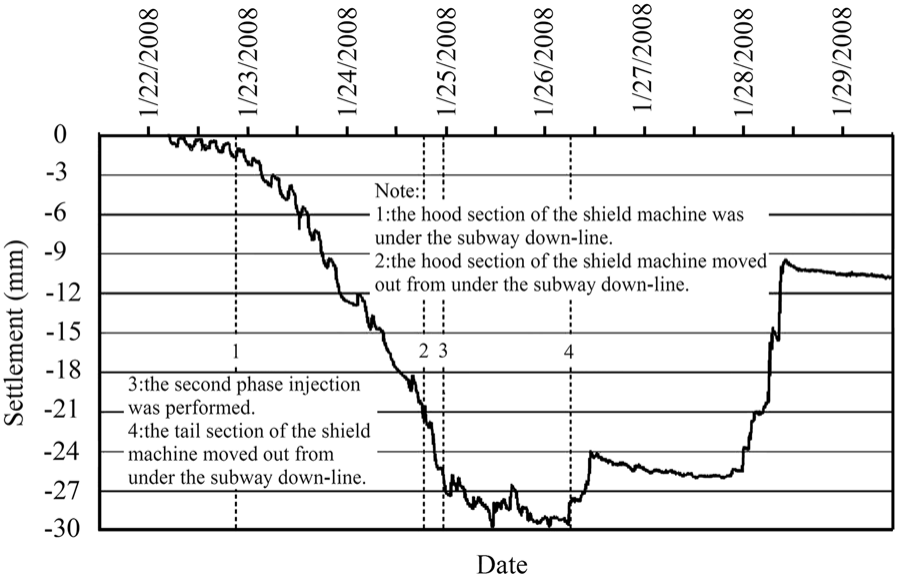
Settlements of Line 8 down-line tunnel during and after building the SXRRC east-line tunnel

**Fig. 26:**
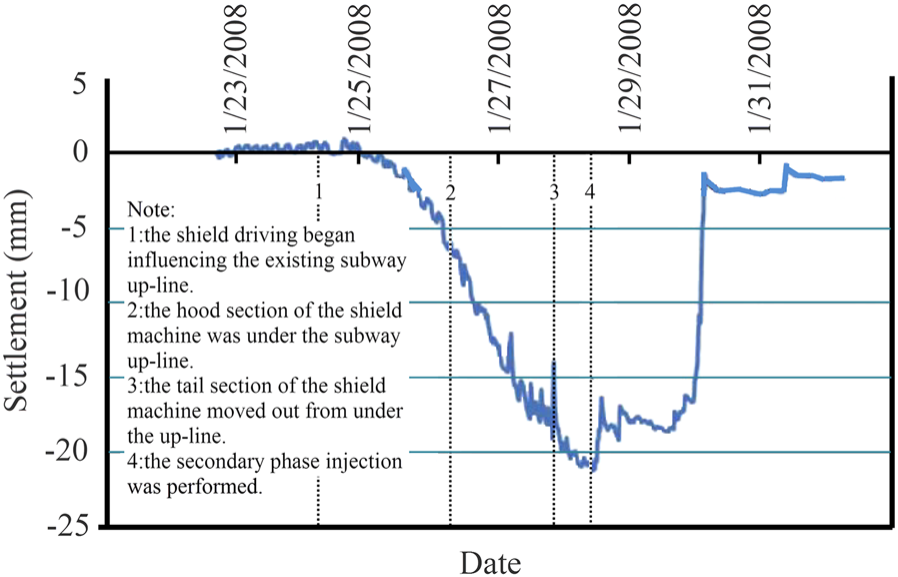
Settlements of Line 8 up-line tunnel during and after building the SXRRC east-line tunnel

**Fig. 27:**
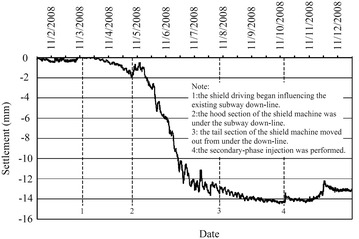
Settlements of Line 8 down-line tunnel during and after building the SXRRC west-line tunnel

**Fig. 28:**
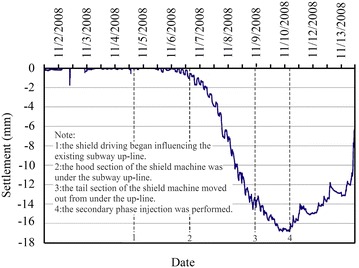
Settlements of Line 8 up-line tunnel during and after building the SXRRC west-line tunnel

The SXRRC east-line tunnel close crossing Line 8 tunnels construction began on January 22, 2008 and ended on January 28, 2008; the SXRRC west-line tunnel crossing Line 8 tunnels construction began on November 4, 2008, and ended on November 11, 2008. The measured movements of Line 8 tunnels during building the SXRRC tunnels are given in Figs. 25, 26, 27 and 28.

**Fig. 29:**
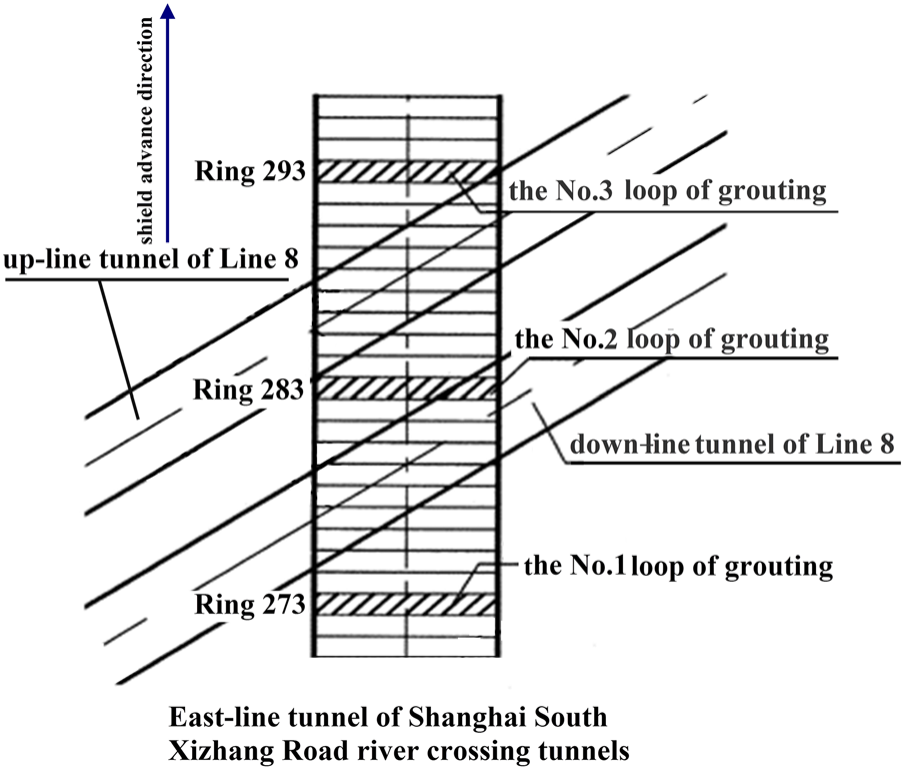
Remedial plan of using grouting in the SXRRC east-line tunnel

**Fig. 30:**
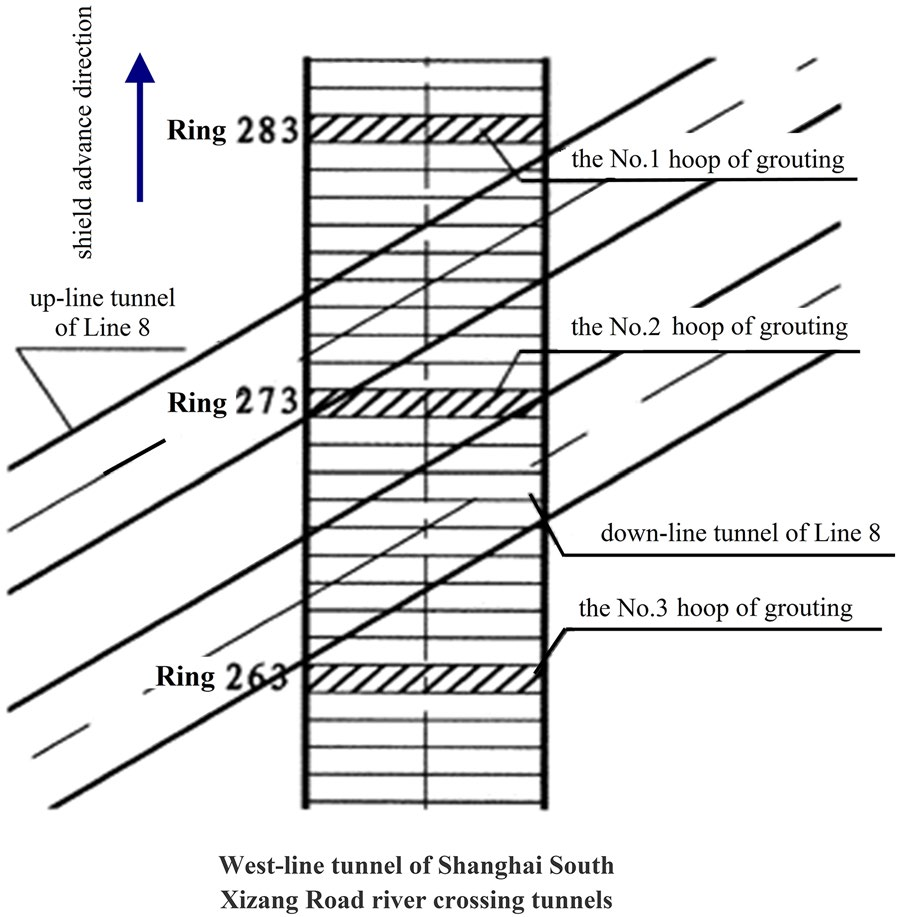
Remedial plan of using grouting in the SXRRC west-line tunnel

Due to pipeline blockages of the simultaneous backfilling grouting in constructing the SXRRC east-line tunnel, the maximum settlements, shown in Figs. 25 and 26, of Line 8 down-line and up-line tunnels once reached 29.68 and 21.25 mm respectively, which had exceeded the predefined allowance of 20 mm. To rectify these large tunnel settlements, a remedial plan of using grouting in the SXRRC east-line tunnel was executed to raise Line 8 tunnels, and the two-component grout of cement and sodium silicate was employed with the initial setting time about 10 s and shrinkage ratio <5 %. The grouting plan mainly included two steps. As given in Fig. 29, three hoops were made by injecting 8 m^3^ grout around the tunnel at the Rings of 273, 283 and 293 at first, and then grouting uniformly at small volume and more points for more times were performed among the three grout hoops. The accumulative grout was more than 60 m^3^ in volume. The settlements of the down-line and up-line tunnels of Line 8 were controlled within 12 and 5 mm respectively. The in-tunnel grouting plan was also employed when constructing the SXRRC west-line tunnel, as given in Fig. 30. The final settlements of Line 8 tunnels after the crossing construction were both below the designed allowance of 20 mm, as presented in Figs. 27 and 28.

### Innovative on-line monitoring tool

An innovative tool i.e. the optical fiber measurement for tunnel movement monitoring, has been developed (Inaudi et al. 1998; Mohamad et al. 2007, 2010; Cheung et al. 2010). The fiber optic sensors can provide a continuous strain distribution of the tunnel linings, whereas conventional strain measurement gauges monitor the strain variations at discrete locations and only provide an approximation for tunnel curvature. Meanwhile, many segmental subway tunnels have been or being constructed worldwide in mainland China in recent years. These tunnels are sensitive to the neighboring construction at the segment joints. The joint movement, decided by the tunnel curvature, is closely related with the tunnel waterproofing. Particularly in the tunnel below-crossing construction, minor joint open of the above segmental tunnel often induce water leakage into the tunnel. This innovative tool of the optical fiber measurement is the preferred choice to get the continuous movement distribution of the disturbed tunnel in the case. Trial applications of this novel technique in monitoring the movement of tunnel linings have been carried out in China (Wang et al. 2013; Huang et al. 2013). But until now, it hasn’t been put into use in the tunnel close crossing construction projects in mainland China mainly due to its higher price. It is expected in the near future the cutting-edge technique will be employed to guarantee safety of the tunnel crossing construction projects in China with the further development of this technology.

## Conclusions

A number of largest cities in China are stepping up the construction of their subway networks, and many new shield tunnels are in close proximity to the existing operational underground subway lines. Key elements of the safety control framework for the new nearby shield tunneling are formulated in a system way on the basis of outlining the completed shield tunnel close crossing projects in mainland China. Discussions are made of the operational tunnel movement, application of remedial grouting and use of the optical fiber measurement for tunnel movement monitoring. In accordance with the statements and discussions, the following is concluded:

1. The shield tunneling method and the staggered single crossing schemes are widely adopted in China when constructing tunnels in close proximity of the existing operational subway tunnels owing to their many advantages.
2. Methodologies for the new nearby shield tunneling progress rapidly with the accumulated experiences and plans for tunnel close crossing construction projects have been set up in the largest cities of China. Many case studies summarized, the six key elements of the safety control framework for the crossing construction plans refer to (1) inspecting the existing operational subway tunnels, (2) deciding allowed movements of the existing tunnels and tracks, (3) simulating effects of the shield tunneling on the existing tunnels, (4) doing the preparation work, (5) monitoring design and information management, and (6) measures and activation mechanism of the countermeasures.
3. Before staring crossing construction, thorough and detailed in-tunnel investigations are conducted, of the functional aspects, mainly focusing on the structural component, and the operational aspects, including track, power supply and other involved systems/appurtenances. Inspection closely intertwined and coordinated with maintenance of the operational subway tunnel, are scheduled and contemplated on a daily basis during the whole process.
4. The allowed horizontal and vertical movements of the existing tunnel determined mainly based on the experiences are in the ranges of 5–20 mm, and recently the allowed movement of 5 mm was often adopted with the increase of the tunnel crossing construction projects. The allowed movements of the track structures are closely related with the types of fasteners and the maintenance plans in different cities.
5. Simulating impacts of shield tunneling on the existing tunnels using FEM or FDM is indispensable to almost all the plans for tunnel close crossing construction projects. But the calculated results are not taken as absolute values, the findings by calculations do provide the crossing projects with directional guidance. The simulation precision is increased solidly lately with the more projects and calculations.
6. Concerning the new nearby shield tunneling, the preparation works highlighted are (1) zoning management of the close crossing projects, (2) suggesting values for key parameters of the shield driving and (3) preparations for the secondary phase grouting work of the shield tunneling.
7. A sensible in-tunnel monitoring design as well as information management is the key to the safeties of both the existing tunnel and the tunnel being built. The combination of the remote reading method and the manual method was often the choice. A joint team consisting of the designer, the builder, the inspection engineer and the owner and the administrator of the existing tunnels are on duty at the jobsite to realize the efficient information management over the period of the close crossing.
8. The widely used measures including countermeasures mainly comprise limiting the speed of trains, grouting work of shield tunneling and grouting work within existing tunnels. The countermeasures are activated during construction according to the predefined triggering criteria, i.e. the attention threshold and alarm threshold. For extremely critical situations, an emergency plan is always prepared in the close crossing projects.
9. Typical close crossing construction cases show that the movement of existing operational subway tunnels in construction can be controlled within 15 mm when excavation diameters are <7 m of the used shield machines. The settlement of tunnels after construction in very soft ground is far larger than that in construction and its duration can last several years. At the same time, using remedial grouting to reduce the long-term settlement of tunnels in very soft clay soils is feasible. Being the more and more segmental tunnels constructed, there is an increasing need of the optical fiber measurement for tunnel movement monitoring in the future in China.

In the safety control framework, the first part provides the foundation for the other five parts. The second part, which finds the criteria to ensure safety of the operational tunnel, is crucial to success of the new tunnel construction. The third part previews the possible results and worst scenarios of the construction, and provides important references for the remaining parts (the fourth, fifth and sixth part). With the last two parts (the fifth and sixth part), the execution plans can be adjusted as needed. The six parts together achieve the safety of the operational subway tunnels.
